# NK Cell Senescence in Cancer: From Molecular Mechanisms to Therapeutic Opportunities

**DOI:** 10.14336/AD.2025.0053

**Published:** 2025-03-13

**Authors:** Zilin Qiu, Zhengrui Li, Cangang Zhang, Qun Zhao, Zaoqu Liu, Quan Cheng, Jian Zhang, Anqi Lin, Peng Luo

**Affiliations:** ^1^Donghai County People's Hospital - Jiangnan University Smart Healthcare Joint Laboratory, Donghai County People's Hospital (Affiliated Kangda College of Nanjing Medical University), Lianyungang, 222000, China.; ^2^Department of Oncology, Zhujiang Hospital, Southern Medical University, Guangzhou, 510282, Guangdong, China.; ^3^Department of Oral and Cranio-Maxillofacial Surgery, Shanghai Ninth People's Hospital, College of Stomatology, Shanghai Jiao Tong University School of Medicine, National Clinical Research Center for Oral Diseases, Shanghai Key Laboratory of Stomatology and Shanghai Research Institute of Stomatology, Shanghai, 200011, China.; ^4^Department of Pathogenic Microbiology and Immunology, School of Basic Medical Sciences, Xi'an Jiaotong University, Xi'an, Shaanxi 710061, China.; ^5^The Third Department of Surgery, the Fourth Hospital of Hebei Medical University, Shijiazhuang, Hebei 050011, China.; ^6^Hebei Key Laboratory of Precision Diagnosis and Comprehensive Treatment of Gastric Cancer, Shijiazhuang 050011, China.; ^7^Big data analysis and mining application for precise diagnosis and treatment of gastric cancer Hebei Provincial Engineering Research Center, Shijiazhuang 050011, China.; ^8^Institute of Basic Medical Sciences, Chinese Academy of Medical Sciences and Peking Union Medical College, Beijing, 100730, China.; ^9^Department of Neurosurgery, Xiangya Hospital, Central South University, Changsha, 410008, Hunan, China.; ^10^National Clinical Research Center for Geriatric Disorders, Xiangya Hospital, Central South University, Hunan, China

**Keywords:** Cell senescence, Immunotherapy, NK cell, Tumor microenvironment (TME), Cytotoxicity

## Abstract

P Natural killer (NK) cells function as crucial effectors in the innate immune response against tumors. Nevertheless, NK cell senescence, characterized by phenotypic and functional changes, substantially compromises their antitumor immune response. This review provides a comprehensive summary of the molecular mechanisms governing NK cell senescence and its implications for cancer immunotherapy. We propose a refined definition of NK cell senescence based on distinct biomarkers, including elevated CD57 expression, reduced cytotoxicity, and altered cytokine secretion. Moreover, we investigate the complex interactions between the tumor microenvironment (TME) and NK cell senescence, highlighting the influence of chronic inflammation, immunosuppressive cytokines, and persistent tumor antigenic stimulation. Additionally, this review underscores the potential utility of senescent NK cells as biomarkers for assessing antitumor efficacy and examines the adverse effects of NK cell senescence on cancer immunotherapy. Lastly, we summarize current approaches to mitigate NK cell senescence, such as gene editing techniques and cytokine modulation, which may enhance the efficacy of NK cell-based immunotherapies. By establishing a comprehensive framework for understanding NK cell senescence within the TME, this review aims to guide future research and the development of innovative therapeutic strategies targeting senescent NK cells to improve cancer immunotherapy outcomes.

## Introduction

Immune senescence refers to the age-related process that leads to progressive deterioration in immune system function. This phenomenon is characterized by alterations in the composition, quantity, and functionality of immune cells [1, 2]. Among the various components of the immune system, Natural Killer (NK) cell senescence contributes significantly to overall immune senescence [3]. NK cell senescence represents a fundamental component of the overall immune aging process [4].

NK cells constitute a vital component of the innate immune system, functioning to recognize and eliminate tumor cells in the body [5-8]. NK cell function is governed by both activating and inhibitory receptors expressed on their surface [9, 10]. These receptors either trigger or suppress NK cell cytotoxic responses through binding to corresponding ligands on target cells [9]. Similarly, various cytokines and chemokines modulate NK cell development and function, enabling these cells to perform distinct immunosurveillance roles across different tissues [10].

In this review, we define NK cell senescence as age-related or pathology-induced alterations in cellular function and phenotype, a process mediated by multiple molecular mechanisms. Senescent NK cells typically display decreased proliferative capacity [11], telomere shortening [9], impaired cytotoxicity [12], dysregulated cytokine secretion patterns [13], and enhanced expression of inhibitory receptors [11]. NK cell senescence not only compromises their cytotoxic function but also modifies their behavior within the tumor microenvironment, thereby diminishing their tumor elimination capacity [14]. These alterations compromise the ability of senescent NK cells to effectively perform immunosurveillance and clearance functions when encountering tumor cells, enabling tumor cells to evade immune surveillance and subsequently facilitating tumorigenesis and disease progression [11].

Recent studies have highlighted several key aspects of NK cell senescence. These include a significant shift in NK cell subsets, characterized by a decrease in immature, cytokine-producing CD56bright subpopulations and an increase in more differentiated CD56dim subsets, which typically express the senescence marker CD57 [15, 16]. Functionally, senescent NK cells exhibit reduced cytotoxicity, decreased production of key cytokines (such as IFN-γ and TNF-α), and impaired responsiveness to activation signals [17, 18]. Surface receptor expression is substantially altered, manifesting as decreased levels of activating receptors (such as NKp30, NKp46, and NKG2D) and increased expression of inhibitory receptors (such as KIR and NKG2A) [18, 19]. The underlying mechanisms driving NK cell immunosenescence are multifactorial, encompassing DNA damage accumulation [20], mitochondrial dysfunction [21], altered micro-environmental cues, and epigenetic modifications [22], including changes in miRNA expression [22, 23]. Emerging evidence suggests that lifestyle interventions (such as exercise and dietary modifications) can modulate NK cell function and potentially mitigate the detrimental impacts of immunosenescence [24]. Targeting specific immune checkpoints and inflammatory pathways may provide promising novel strategies to rejuvenate senescent NK cells and enhance their anti-tumor immunity. Senescent NK cells severely compromise immune surveillance [22, 25], and senescence disrupts the balance between NK cell subsets, thereby undermining the overall cytotoxic capacity of the NK cell population and accelerating tumor progression [24]. Understanding the intricate molecular mechanisms of NK cell senescence is crucial for developing effective immunotherapeutic approaches to combat cancer and other age-related diseases.

Despite significant progress in the study of NK cell senescence mechanisms in recent years, many key issues still remain to be addressed. First, the precise definition of NK cell senescence remains controversial and continues to lack scientific consensus [26]. Second, the specific molecular mechanisms underlying NK cell senescence, particularly the functional roles of various surface markers and intracellular signaling pathways, have not been fully elucidated. Furthermore, research investigating the induction of NK cell senescence within the tumor microenvironment remains limited. Many questions remain unanswered regarding how cellular and molecular factors within the tumor microenvironment—such as myeloid-derived suppressor cells (MDSCs), regulatory T cells (Tregs), and inhibitory cytokines (e.g., TGF-β, IL-6)—influence NK cell senescence through complex interaction networks [27]. Moreover, in-depth studies examining the specific role of NK cell senescence in modulating sensitivity to antitumor drugs are lacking. The impact of senescent NK cells on the efficacy of immune checkpoint inhibitors and chimeric antigen receptor (CAR)-NK cell therapies also warrants further investigation, as these critical relationships remain poorly defined [28]. Additionally, strategies to effectively mitigate NK cell senescence through targeted gene editing or cytokine modulation, thereby enhancing their antitumor and antiviral functions, warrant further exploration. Cytokines such as IL-2, IL-15, and IL-21 have demonstrated promising potential in attenuating NK cell senescence; however, their precise molecular mechanisms necessitate further investigation [29].

This review thoroughly examines the definition, characteristics, and molecular mechanisms of NK cell senescence. NK cell senescence manifests as altered surface markers and functional impairment, characterized by reduced expression of activating receptors (such as NKG2D, NKp30), increased expression of inhibitory receptors (such as KIRs, NKG2A) [9, 30, 31], and KLRG1-mediated activation of the AMPK signaling pathway, which subsequently leads to suppressed telomerase activity and diminished cytotoxicity. Due to the lack of a clear definition of the "healthy aged" NK cell compartment [11, 32] and insufficient in-depth research on how NK cell senescence affects the expression and function of activating and inhibitory receptors across different subsets, future studies are essential to further investigate NK cell senescence mechanisms and define the precise characteristics of a senescent NK cell compartment [32], thus enhancing our understanding of its role in immunosenescence [3, 33].

In this review, we examine the mechanisms that contribute to NK cell senescence within the tumor microenvironment (TME), with a particular emphasis on the DNA damage response pathway, persistent tumor antigenic stimulation, and the influence of suppressor cells and cytokines. Chronic inflammation and immunosuppressive cytokines within the TME play a significant role in the development of NK cell senescence. Immunosuppressive cytokines impair NK cell function through various mechanisms, including inhibiting proliferative capacity, reducing cytokine production, and modulating receptor expression profiles [34]. Furthermore, chronic tumor antigenic stimulation functions as a crucial driver of NK cell senescence [35]. Notably, chronic viral infections, particularly human cytomegalovirus (HCMV), have been shown to be strongly associated with NK cell senescence. HCMV infection not only elevates the proportion of CD57+ CD56dim NK cells but also impairs both the cytotoxic potential and proliferative capacity of NK cells [10].

Furthermore, this review examines the role of senescent NK cells in tumor immunity, evaluates their potential as biomarkers for monitoring antitumor efficacy, and analyzes the inhibitory effects of their functional alterations on antitumor immunity. Finally, this review summarizes current strategies for attenuating NK cell senescence. Gene editing techniques, such as CRISPR-Cas9, have demonstrated significant potential in modifying NK cell genes to decelerate their senescence [36]. Studies have demonstrated that hTERT overexpression can restore NK cell function following expansion and substantially decelerate senescence progression [37]. These findings not only provide a promising approach for NK cell-based immunotherapy but also establish a foundation for elucidating the molecular mechanisms underlying NK cell senescence.

First, this review dissects the molecular mechanisms of NK cell senescence, elucidating the roles of critical surface markers and their associated signaling pathways in the senescence process. Additionally, it analyzes the key factors that induce NK cell senescence in the tumor microenvironment, revealing their contributions to tumor development and progression, while highlighting directions for future research. Furthermore, this article evaluates the complex role of senescent NK cells in tumor immunity and emphasizes their utility as potential biomarkers for monitoring antitumor therapeutic efficacy, thereby providing novel perspectives for clinical applications. Finally, it summarizes current approaches to delay NK cell senescence and outlines future research directions, such as identifying specific molecular markers of senescent NK cells, thoroughly investigating their interactions with the tumor microenvironment, antitumor drug sensitivity, and intratumoral microbiome. These investigations aim to provide theoretical foundations and scientific evidence for developing more refined antitumor immunotherapeutic strategies.

## 1. Definition and characterization of NK cell senescence

**Figure 1.**
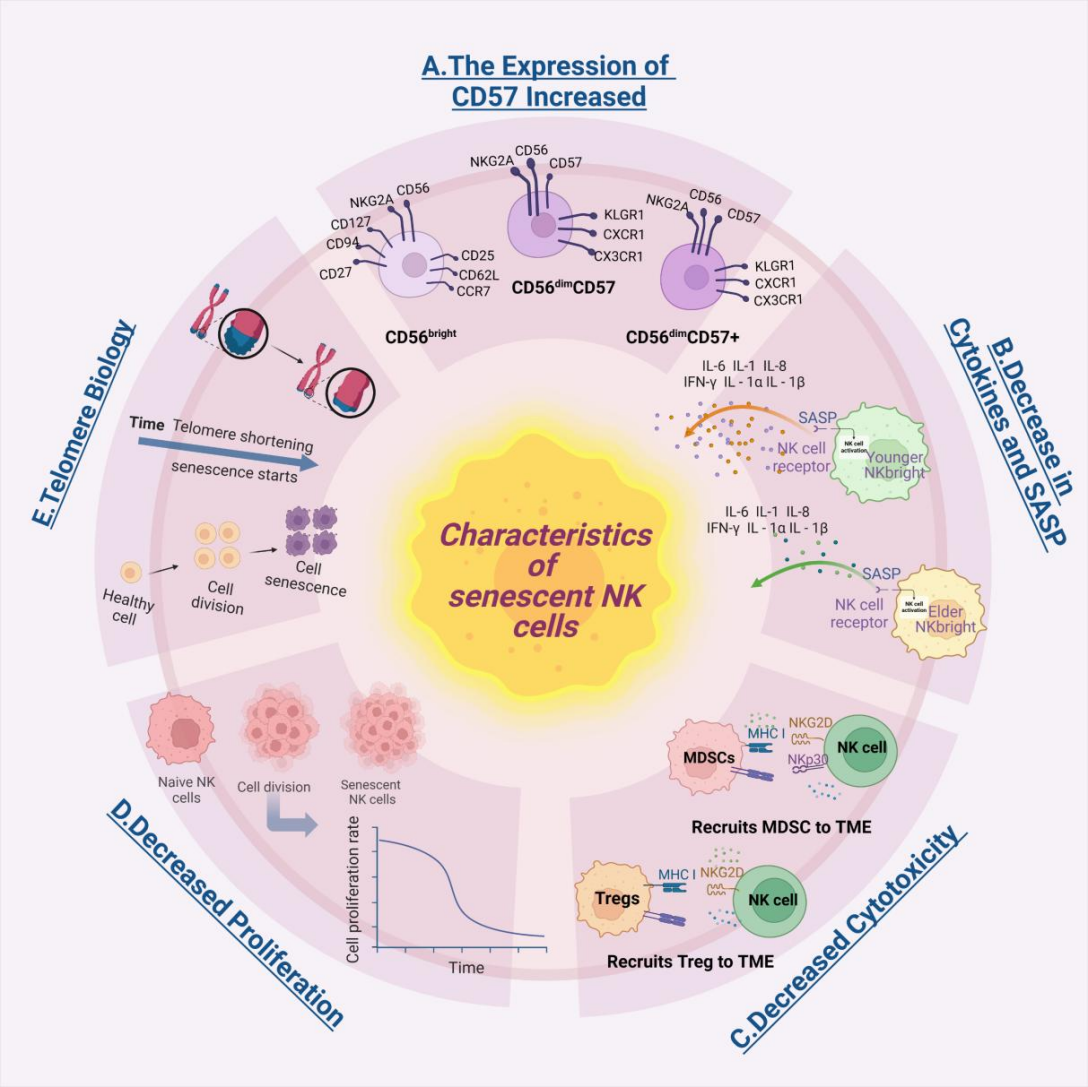
**Characterization of senescent NK cells**. The image depicts five major characteristics of senescent NK cells. A) Increased expression of the senescence marker CD57, which serves as a key indicator of NK cell aging. The schematic demonstrates the differentiation process from CD56bright to CD56dimCD57- to CD56dimCD57+ NK cells, emphasizing the differences in major markers between these distinct cell populations. The transition from CD56bright to CD56dim NK cells is characterized by more significant changes in surface markers, while the progression from CD56dimCD57- to CD56dimCD57+ NK cells primarily involves an increased intensity of CD57 expression, indicating advancing senescence. B) Release of the senescence-associated secretory phenotype (SASP), a hallmark of cellular senescence. Cytokine production is significantly reduced in aged CD56bright NK cells compared to their young counterparts, contributing to the altered SASP profile. C) Reduced cytotoxicity, which impairs tumor cell elimination capacity. MDSCs and Tregs synergistically inhibit NK cell function through distinct yet complementary mechanisms. MDSCs inhibit NK cell function by blocking the NKp30 and NKG2D receptors on the NK cell surface and interfering with the NK cell activation process, thereby suppressing cytolytic activity. Concurrently, Tregs further suppress NK cell cytotoxicity by promoting TGF-β/NKG2D interactions. Consequently, MDSC- and Treg-induced NK cell immune senescence likely plays a crucial role in cancer progression. D) Decreased cell proliferation, limiting expansion capacity. For therapeutic applications, researchers have significantly expanded NK cells using in vitro culture techniques, though senescent NK cells show diminished expansion potential. Progressive telomere shortening results from an inherent deficiency of DNA polymerase in replicating chromosome ends. These shortening compromises cell division, with the proliferative potential of NK cells significantly diminishing as telomeres reach a critical length, known as the Hayflick limit. E) Telomere shortening and telomerase deficiency, key molecular determinants of senescence. When telomeres shorten to a critical length, the cell cannot maintain normal DNA replication due to insufficient telomerase activity, subsequently leading to senescence or apoptosis. This figure was generated using tools provided by Biorender.com (accessed on May 30, 2024). SASP, senescence-associated secretory phenotype; MDSCs, myeloid-derived suppressor cells; Tregs, regulatory T cells.

NK cell senescence is characterized by various features, including increased cell surface CD57 expression (as shown in Figure 1), decreased cytotoxicity, altered cytokine production, and a diminished capacity to regulate the immune response with advancing age [38-42]. However, a universally accepted definition of NK cell senescence has yet to be clearly established. Our proposed definition of NK cell senescence is derived from a comprehensive synthesis of current literature describing the hallmarks of this phenomenon. The identification of these characteristics provides novel insights into the mechanisms underlying NK cell senescence and its potential impact on antitumor immunity.

Immunosenescence is characterized by an age-associated decline in immune system function [2, 43], consequently resulting in a diminished capacity for effective antitumor responses [44]. Although functional impairments can occur with senescence, it is crucial to distinguish between immunosenescence and physiological senescence, as some research perspectives erroneously equate these two distinct processes [45, 46]. Furthermore, the immune system alterations observed in young and middle-aged cancer patients bear striking similarities to the processes of "premature immunosenescence" or "cancer-induced immune-senescence" typically associated with healthy older individuals [10]. Although T cells exhibit the most prominent functional decline in aging-associated immune response alterations, significant age-related immunological changes have also been observed in the phenotype and function of NK cells. This finding highlights that the role of NK cells, alongside T cells, in the cellular immune response undergoes significant alterations during senescence, a phenomenon that has crucial implications for a more comprehensive understanding of how aging impacts the overall immune system landscape [47, 48]. Age-related NK cell immune senescence is associated with higher rates of viral infection and cancer induction [13], thereby emphasizing the critical importance of delaying NK cell senescence as a potential strategy to reduce cancer incidence. Senescent NK cells may exhibit reduced efficiency in eliminating tumor cells due to their impaired cytotoxic function [26, 37, 49], which may compromise anti-tumor surveillance. Additionally, NK cell senescence alters cytokine production, potentially compromising the synergistic effects of senescent NK cells with other immune cells, such as T cells [50], and disrupting the regulation of the immune response [42] and the tumor microenvironment (TME) [14]. Therefore, it is crucial to refine the definition of NK cell senescence and thoroughly investigate its molecular and functional characteristics along with its clinical implications, as these insights will be invaluable for developing novel therapeutic strategies and improving the health of the elderly population.

### 1.1 Definition of senescent NK cells

There is currently no universally accepted or standardized definition to describe the senescence of NK cells. In response to this lack of consensus, researchers have employed specific biomarkers to characterize senescent NK cells, with increased CD57 expression being commonly used in current research protocols. No clearly established phenotypic markers exist to definitively define NK cell senescence in the oncology field [29]. Several attempts have been made to define NK cell senescence. For example, changes in the composition, phenotype, and function of the circulating NK cell pool associated with advancing physiological age have been characterized as immunosenescence of NK cells [4]. Additionally, studies have established that NK cell immunosenescence is characterized by impaired NK cell cytotoxic and immunoregulatory function [13]. Increased activity of senescence-associated β-galactosidase (SA-β-gal), a widely used biomarker of senescence that indicates increased lysosomal biosynthesis in senescent cells, has limited applicability to NK cell senescence assessment. While SA-β-gal activity correlates with general cellular senescence [11], it lacks specificity to NK cell senescence [51] and therefore cannot serve as a definitive marker for identifying senescent NK cells.

### 1.1.1 Aging Markers Increased CD57 expression

CD57 is a terminally sulfated glycoantigenic epitope that specifically marks highly differentiated and senescent NK cells [39]. In a study examining the effect of exercise on acute senescent lymphocyte counts, researchers identified CD57+ NK cells as displaying a senescence-like phenotype [52]. Among the numerous features that characterize NK cell senescence, CD57 expression serves as a general marker of reduced proliferative capacity, extensive cell division history, and shorter telomeres [31, 53, 54]. In CD57- subpopulation-derived NK cell clones, which may acquire CD57 expression in culture, the proportion of CD57+ cells negatively correlates with clonal lifespan [39], indicating a positive association with NK cell replicative senescence. This evidence confirms that CD57 expression serves as a marker of reduced proliferative capacity in NK cells, a key characteristic of NK cell senescence. This phenomenon may be associated with the senescence of NK cells observed in culture [39]. Further studies have demonstrated that during terminal differentiation of NK cells, NKG2A expression is progressively lost, whereas the expression of inhibitory killer-cell immunoglobulin-like receptors (KIRs) and CD57 progressively increases [31]. This series of changes represents a gradual shift in both the immunophenotype and function of NK cells during the aging process. Therefore, this review will examine the role of CD57 in NK cell senescence, specifically focusing on how it emerges during NK cell differentiation and its establishment as a definitive marker of NK cell senescence.

**Figure 2.**
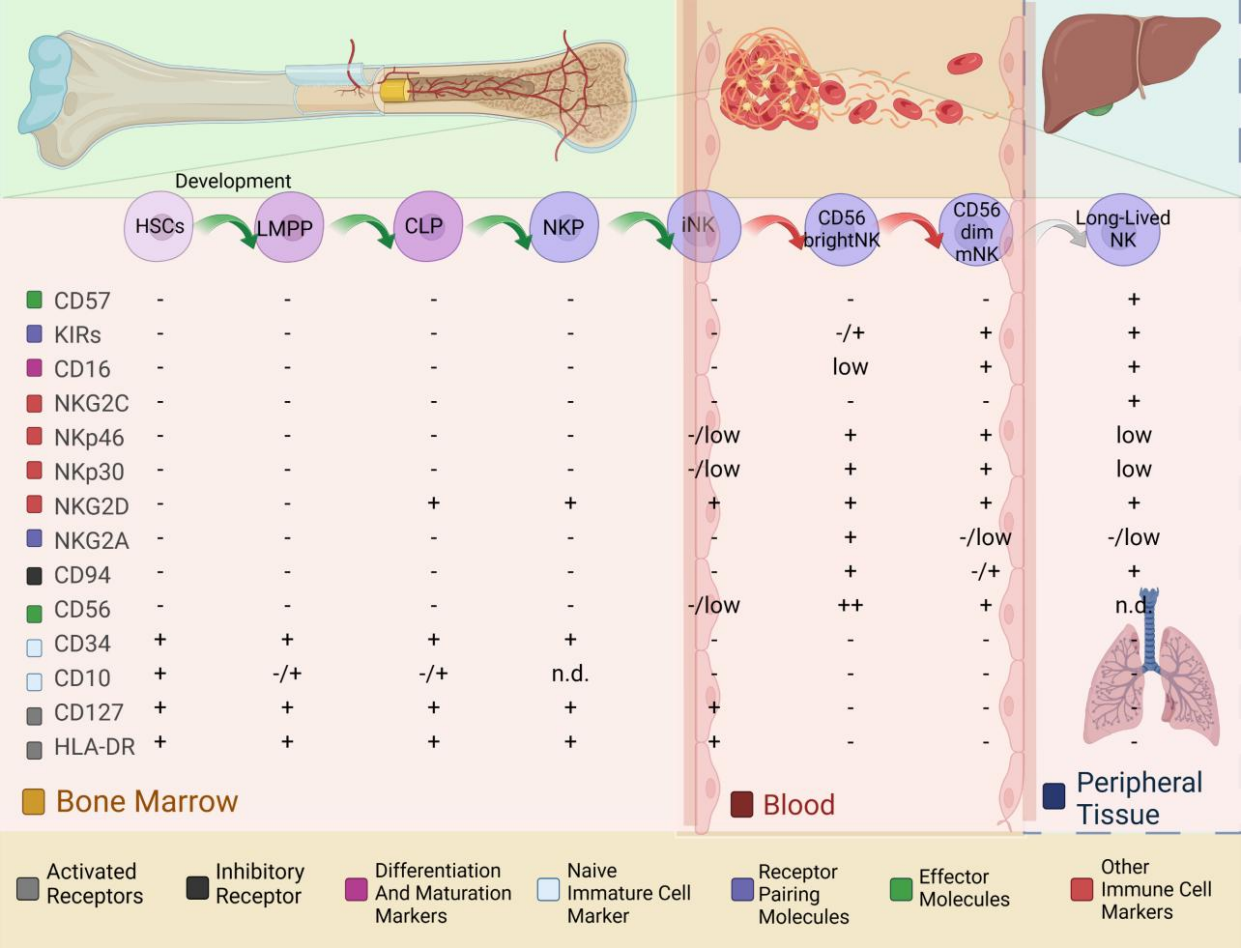
**Linear model of human NK cell development**. This figure presents a schematic representation of the different stages of NK cell differentiation in human bone marrow and secondary lymphoid tissues, depicting a linear pathway from left to right. NK cell differentiation commences with hematopoietic stem cells (HSCs) in the bone marrow, which initially develop into lymphoid-primed multipotent progenitors (LMPPs) and subsequently differentiate into common lymphoid progenitors (CLPs). NK progenitors (NKPs) then develop into immature NK cells (iNKs) that enter the bloodstream, where they further differentiate into mature NK cells (mNKs). Mature NK cells are categorized into two main subsets: CD56bright and CD56dim, with CD56dim NK cells predominantly expressing CD16. Long-lived NK cells can be identified by elevated CD57 expression and persist for extended periods in peripheral tissues, particularly the liver and lungs. Flow cytometric analysis has revealed that, with the exception of CD57, the gradual loss of NKG2A expression is accompanied by the sequential acquisition of inhibitory killer cell immunoglobulin-like receptors (KIRs). The color blocks in the figure categorize the markers into seven main groups, illustrating the common patterns of marker expression changes during NK cell development. This figure was created using visualization tools provided by Biorender.com (accessed on May 30, 2024). Abbreviations: HSC, hematopoietic stem cells; LMPP, lymphoid-primed multipotent progenitor cells; CLP, common lymphoid progenitor cells; NKP, NK progenitor cells; iNK, immature NK cells; mNK, mature NK cells; NCR, natural cytotoxicity receptor; KIR, killer cell immunoglobulin-like receptor. Expression levels are designated as "+" (expressed), "-" (no expression), "low" (low expression), and "n.d." (undetermined).

A hallmark of cellular senescence is the upregulation of CD57, and the dynamic changes in CD57 expression across NK cell subpopulations are critical for understanding the senescence characteristics of NK cells. Before exploring this phenomenon further, a concise overview of the developmental differentiation process of NK cells and their subpopulations will enhance our understanding of the dynamic changes in CD57 expression. NK cell differentiation originates from hematopoietic stem cells (HSCs) in the bone marrow [55, 56], which initially develop into common lymphoid progenitor cells (CLPs) [9, 36, 57, 58] and subsequently differentiate into NK cell progenitors (NKPs) [36, 59]. NKPs then give rise to immature NK cells (iNKs) [60], which subsequently differentiate into mature NK cells (mNKs). The developmental trajectory of NK cells and their tissue distribution are illustrated in Figure 2. The predominant mNK subset is typically identified as CD56dim NK cells [36]. The sequential acquisition of CD56, CD94, and the Killer C-type lectin receptor CD161 expression has been established as a marker for the transition from NKPs to mature NK cells [61].

The human mature NK cell population is subdivided according to the expression of the surface markers CD56 and CD16: the cytotoxic population CD56dim / CD16pos (CD56dim), which accounts for 90% of circulating NK cells; and the regulatory NK cell subsetCD56bright / CD16neg (CD56bright), which produces a large number of pro-inflammatory cytokines, such as Interferon IFN-γ and TNF-α [62], For instance, IFN-γ coordinates enhanced anti-tumor responses by regulating dendritic cell antigen presentation and triggering CTL activation [63]. These cytokines are instrumental in the anti-infective and anti-tumor immune responses through the activation of dendritic cells [64]. A study demonstrating the terminal differentiation steps of NK subpopulation development supports the sequential linear differentiation model of NK subpopulation development (CD56bright→ CD56dimCD57-→CD56dimCD57+) [9].

Senescence of NK cells has been observed during human aging, with senescent NK cells predominantly belonging to the CD56dim NK subpopulation [50]. NK cells exhibit distinct phenotypic changes during senescence, which reflect the evolution of their function and state. Studies have shown that aging causes an imbalance in NK cell subsets, specifically manifested as an increased proportion of CD56bright subsets with lower cytotoxicity and a concomitant decrease in CD56dim subsets with higher cytotoxicity, thereby reducing overall cytotoxic activity [16]. However, contradictory findings from another study reported an increase in the cytotoxic CD16+CD56dim NK cell subpopulation and a decrease in the immunomodulatory CD16-CD56bright NK cell subpopulation, along with higher CD57 expression [65]. Potential causes of these inconsistent research findings warrant further investigation, including differences in study populations and variations in detection methodologies. During the aging process, CD56dim NK cells continue to differentiate, progressively increasing the expression of CD57 and CD16 markers, as well as activating receptors such as NKG2D [18]. Interestingly, no significant difference has been observed between CD57-positive and CD57-negative NK cells regarding cytokine production and cytotoxicity against target cells. These findings highlight the complexity of phenotypic and functional changes that occur during NK cell differentiation. The specific phenotype of senescent NK cells has not been fully elucidated, and the functional consequences of NK cell senescence necessitate further investigation.

### 1.1.2 Other markers of NK cell senescence

In addition to CD57, the combined expression patterns of KIRs, NKG2A, NKG2C, NKp30, NKp46, NKp44, CD16 (FcγRIII), CD11b, and CD27 have all been correlated with NK cell senescence, with CD57, KIRs, NKG2A, and NKG2C being more widely used [9, 31-33, 64, 66]. The unidirectional differentiation marker expression sequence from CD56bright to CD56dim progresses from NKG2A+ NKG2C-, to NKG2A- NKG2C-, and finally to NKG2A- NKG2C+ [67]. The progressive loss of NKG2A expression is accompanied by the sequential acquisition of inhibitory KIRs and the emergence of CD5 [1, 31, 68]. Moreover, NKG2A and KIR expression are complementary [69, 70]. However, each of these widely used markers has its own advantages and limitations: the loss of NKG2A may be a reversible event, while the acquisition of KIRs and expression of CD57 are irreversible both *in vitro* and *in vivo*. Nevertheless, it has been demonstrated that all CD56dim NK cells differentiate according to a fixed linear scheme [31]. In the context of tumor development, the expression levels of NKG2A and NKG2C reflect the inhibition or activation status of NK cells [1, 4, 71-73], thereby providing useful indicators for studying the regulatory mechanisms of NK cells; however, the function of NKG2A and NKG2C is dependent on the expression of their ligands, such as HLA-E molecules, which may be affected by a variety of factors, thereby limiting the analyses based on these markers [38, 74]. Therefore, the expression of a single marker is not sufficient to fully reflect the functional status of NK cells. Careful consideration of the choice and combination of markers, along with their relevance and limitations in different study contexts, should be thoroughly evaluated during experimental design, as this is essential for improving the accuracy and reliability of the study.

NK cells regulate their activity through a series of surface receptors, with NKG2A and KIRs representing two key classes of major histocompatibility complex class I (MHC-I)-specific inhibitory receptors [64]. These receptors play crucial roles in the regulation of NK cell function. Compared to KIR-NKG2A+ NK cells, the KIR+NKG2A- NK cell subpopulation exhibits a distinct expression pattern of maturation-associated markers, characterized by decreased levels of CD62L and CD27 and increased expression of CD57 [39, 26, 65]. NKG2A is predominantly expressed in the early stages of NK cell development, whereas the acquisition of KIRs signifies the transition of NK cells to a mature state [65]. As NK cells mature, CD57 expression increases, a phenomenon regarded as a marker of NK cell senescence and diminished function [33]. These changes strongly support a linear NK cell differentiation process, based on transcriptional and functional characterization of equivalent subpopulations, as well as *in vivo* reconstitution experiments. This process not only reveals important molecular events in the NK cell life cycle, but also provides key clues for understanding the decline of NK cell immunosurveillance in the elderly. Future studies should further explore the specific mechanisms by which NKG2A and KIR contribute to the functional senescence of NK cells and their potential applications in disease control.

Senescence-associated changes in NK cells may represent either genuine immune senescence, adaptive immune alterations, or individual developmental evolution of the NK cell lineage [31]. Markers of NK cell senescence are not absolute; for example, CD57, though frequently observed in senescent NK cells, demonstrates high sensitivity but low specificity, rendering it imprecise as a standalone marker for NK cell senescence [11]. Therefore, there is an urgent need for multi-omics analyses that integrate genomics, transcriptomics, proteomics, and metabolomics data to identify more reliable and clinically relevant combinations of NK cell senescence markers that can accurately characterize the senescent phenotype. Based on current evidence, our perspective on defining NK cell senescence is as follows: first, the primary requirements for marker combinations are ease of detection, high specificity, and high stability in diverse clinical contexts. Second, in-depth investigation of "healthy aging" NK cell characteristics is crucial, as this helps distinguish pathological senescence from natural aging processes, thereby enabling a more comprehensive evaluation of specific traits and markers that collectively define senescent NK cells. Finally, exploring personalized approaches to assess NK cell senescence is essential, including developing non-invasive detection methods based on peripheral blood samples to better monitor the senescence process and guide individualized treatment strategies, thereby providing a robust theoretical basis for developing more effective anti-tumor therapeutic interventions. In summary, research on NK cell senescence still has a broad scope for exploration. To date, numerous studies have focused on characterizing and quantifying the senescence process of NK cells through various biomarkers and functional assays, underscoring the importance of thoroughly exploring the characteristics of senescent NK cells in the context of cancer immunology.

### 1.2 Changes in senescent NK cell characteristics

The complexity of NK cell senescence involves multiple interrelated mechanisms that are critical to this process, including telomere shortening and a decline in cellular proliferative capacity [18, 75]. These alterations reflect the transition of NK cells from reduced proliferative potential to senescence, highlighting telomere length as a critical indicator for monitoring NK cell senescence and functional decline.

### 1.2.1 Decreased cell proliferation

NK cell senescence is typically characterized by diminished effector function and proliferation [11, 26]. Research suggests that during senescence, long-lived, resting state, or mature NK cells accumulate preferentially over their immature precursor cells. Senescence is invariably accompanied by reduced cell proliferative potential in lymphocytes [76], and NK cells are no exception [77, 78]. Numerous research groups have employed in vitro expansion of NK cells to obtain sufficient quantities of these cells for patient treatment. The limited retention time of in vitro expanded NK cells in patients suggests that their enhanced in vitro proliferation and subsequent poor in vivo persistence may result from continuous proliferation leading to NK cell senescence, a phenomenon observed in K562-based artificial antigen-presenting cells (aAPC) [26]. CD57+ NK cells demonstrate reduced proliferation in response to cytokine or target cell stimulation, whereas CD57- NK cells exhibit greater proliferative capacity [11], confirming that CD57 expression has long been associated with a progressive loss of proliferative capacity [39]. The characteristics of NK cell senescence are interconnected and complementary, collectively defining the senescent NK cell phenotype.

### 1.2.2 Telomere shortening and telomerase deficiency

Telomeres are specialized DNA-protein structures that protect the ends of linear chromosomes. During the process of generalized cellular senescence, telomeres progressively shorten with each successive cell division [79]. This phenomenon occurs primarily due to the insufficient activity of telomerase, the enzyme responsible for maintaining telomere structure and function [80]. Therefore, telomere shortening is widely regarded as a "molecular clock" that indicates potential cellular senescence and serves as a reliable marker of cellular senescence [81]. When telomere length diminishes to a critical threshold, cells become incapable of replication due to telomerase deficiency and subsequently undergo either senescence or programmed cell death [82, 83]. Similarly, lymphocyte telomeres inevitably shorten during cellular proliferation and differentiation, potentially driving highly differentiated cells of these lineages toward cellular senescence as a consequence of telomere attrition [33]. Experimental evidence has established that immature CD56dim NK cell subpopulations possess longer relative telomere lengths compared to more mature CD56bright NK cells [31, 33, 42, 84]. Considering that organismal aging must also be taken into account, this telomere attrition process has been proposed as a potential underlying mechanism for lymphocyte aging [84]. Consequently, telomere length analysis has been employed to delineate NK cell subpopulations and assess age-related changes, thereby providing valuable insights into NK cell differentiation and senescence processes [33]. Telomere length serves as a critical biomarker that accurately reflects both the replicative history and senescence status of cells [84]. Emphasizing the significance of telomere length in assessing NK cell senescence establishes a foundation for the comprehensive characterization of senescent NK cells. Telomere maintenance is essential for the sustained survival and proliferative capacity of actively dividing cells [39]. Telomere attrition in NK cells is directly associated with impaired end replication by DNA polymerase, consequently leading to reduced cell division potential [85]. Preserved telomere length and adequate telomerase expression enable NK cells to undergo additional cell divisions and effectively delay the onset of replicative senescence [85], Given that telomere length is significantly reduced in adult NK cells and strongly associated with proliferation-induced senescence [77], it is crucial to investigate the impact of NK cell expansion systems on telomere dynamics, which serve as a critical indicator of NK cell proliferative capacity. Furthermore, in vitro-expanded NK cells exhibit significantly elevated telomerase expression, which subsequently results in increased telomere length [77]. These findings establish a direct connection between telomere length and the diminished proliferative potential of senescent NK cells, thereby highlighting critical changes in the immune functionality of senescent NK cells.

## 1.3 Altered function of senescent NK cells

### 1.3.1 Reduced cytotoxicity function

NK cell senescence is typically characterized by a significant decline in effector function [11, 24, 49, 86]. In healthy young adults, NK cells are considered to be short-lived cells [9, 87], and they maintain relatively quiescent functional properties throughout their life cycle [31]. However, analysis of NK cell homeostasis in elderly donors has revealed a high proportion of memory-characterized, long-lived NK cells [9, 87], which may be associated with an increased proportion of CD56dim NK cells [88]. Notably, cytotoxic changes in NK cells during aging are organ-specific. Studies have shown that in a mouse model, NK cells accumulate in neurogenic microenvironments, such as the hippocampal dentate gyrus in the CNS, where the binding of the activating receptor NKG2D on NK cells to senescent DCX+ cells expressing RAE-1 leads to DCX+ cell killing by NK cells and subsequent cognitive function decline [89]. Myeloid-derived suppressor cells (MDSCs) may inhibit NK cell function by blocking the NKp30 (NCR3) and NKG2D receptors expressed on NK cells. Regulatory T cells (Tregs) also inhibit NK cell function via the TGF-β/NKG2D complex. Thus, MDSC/Treg-induced immune senescence of NK cells may impair the clearance of senescent cells, tumor cells, and various pathogens under aging and inflammatory conditions [40]. In addition, human NK cells have been reported to exhibit characteristics of immunosenescence in acute myeloid leukemia, thereby compromising immune surveillance [40]. Notably, telomerase reverse transcriptase (TERT) overexpression sustains both the growth and functional preservation of NK cells well beyond the senescence-imposed tipping point [90]. The role of TERT in NK cell senescence will be discussed in detail in subsequent sections.

### 1.3.2 Release of inflammatory cytokines (SASP)

CD107a degranulation, IFN-γ, and granzyme B production primarily define the cytotoxic capacity of NK effector function, rather than toxicity or inflammation per se [26]. Elderly-like NK cells demonstrate diminished responsiveness to IL-2 stimulation with respect to IFN-γ production. Alterations in IFN-γ secretion may affect various immune cells, as IFN-γ promotes Th1 differentiation, enhances macrophage function, stimulates leukocyte migration to infection sites, and induces upregulation of major histocompatibility complex expression, ultimately facilitating improved recognition of infected or malignant cells by T cells [50]. In contrast to NKdim cells, NKbright cells exhibit senescence-related declines in total number, phenotypic characteristics, and functional capacity. Research has demonstrated that aged NKbright cells produce significantly lower levels of cytokines, including IFN-γ, MIP-1α, and IL-8, compared to their younger counterparts [13]. Furthermore, senescent NK cells have been shown to secrete elevated levels of pro-inflammatory cytokines. The secretion of multiple inflammatory factors and immunomodulators by senescent cells is known as the senescence-associated secretory phenotype (SASP) [10, 14]. In NK cells, these pro-inflammatory factors include interleukin-6 (IL-6), interleukin-1 alpha (IL-1α), and interleukin-1 beta (IL-1β). Upon CD158d binding, increased protein secretion and gene upregulation were detected in both peripheral blood and metaphase NK cells [14]. The molecular switch that triggers SASP in NK cells is the DNA damage response (DDR) signaling pathway initiated by CD158d (discussed below). SASP exerts dual effects on the surrounding tissue microenvironment. SASP promotes tissue repair during wound healing and, under physiological conditions, recruits immune cells to eliminate senescent cells and prevent tumorigenesis. However, under certain conditions, SASP can contribute to tumor progression and age-related tissue dysfunction [10, 14].

### 1.3.3 Chemotactic inhibitory immune cells in TME

In the TME, persistent inflammation facilitates the chemotaxis of immunosuppressive cells through various mechanisms. These immunosuppressive cells include MDSCs, which are further categorized into monocytic MDSCs (M-MDSCs) and polymorphonuclear MDSCs (PMN-MDSCs) [91]. Additionally, Tregs, regulatory B cells (Bregs), regulatory natural killer cells (NKregs), regulatory dendritic cells (DCregs), M2 macrophages, and type II natural killer T (NKT) cells also constitute critical components of the complex immunosuppressive network [92]. These cells function synergistically to establish a complex immunosuppressive network within the TME. The persistent inflammatory state within the TME not only stimulates MDSC production but also augments the plasticity of immune cells, enabling their phenotypic diversification and potential transdifferentiation into other immunosuppressive cell types. This process potentiates the differentiation and function of immunosuppressive cells, thereby amplifying the immunosuppressive properties of Tregs and Bregs. Concurrently, it attenuates the immunoreactivity of effector T cells and B cells, ultimately culminating in the development of an immunosenescent phenotype.

When investigating the relationship between NK cell senescence and the induction of suppressive immune cells, we first examine the complex interactions between inflammation and immunosuppressive networks. We hypothesize that NK cells, as pivotal components of the innate immune system, may induce the formation of suppressive immune cells as a consequence of senescence-associated functional alterations, reflecting the profound effects of senescence on the entire immune microenvironment. However, additional rigorous experimental evidence is necessary to substantiate this hypothesis. In the following section, we further elucidate the relationship between inflammation and immunosuppressive networks. Chronic inflammation in the TME initiates immunosuppression, facilitating tumor cell evasion of immune surveillance [93]. These findings indicate that NK cell senescence is intrinsically connected to the decline in immune surveillance and the accumulation of senescent cells in tissues. This accumulation further potentiates the function of the immunosuppressive network, thereby modulating the activity and differentiation of suppressor immune cells, which consequently may facilitate tumor growth and metastasis.

### 1.3.4 Reduced capacity to regulate the immune response

Recent studies have revealed that alterations in the chromosomal accessibility of NK cells are associated with the activation of inflammatory pathways and the impairment of the immune response [41]. Notably, in the subpopulation of NK cells, significant changes in key transcription factors occur during senescence, as demonstrated by an increased chromatin accessibility of repressive receptor genes and a decreased chromatin accessibility of activating receptors. These alterations may impair the ability of NK cells to eliminate virus-infected cells [41]. Further comparisons revealed that, compared to NKdim cells, NKbright cells exhibit a significant reduction in number [22], phenotype, and function due to senescence, primarily attributed to their diminished capacity for immune regulation, attenuation of inflammatory responses, and stimulation of adaptive immunity [13]. These alterations in NK cells render them an ideal model for investigating intrinsic and adaptive immune responses in the context of aging [48].

### 1.4 Abnormal NK cell expansion can lead to NK cell senescence

*In vitro* expansion is widely recognized as an effective method to increase NK cell numbers; however, this artificial expansion process may induce cellular senescence, manifested by alterations in telomere length and compromised immune function. Researchers have achieved significant amplification of NK cells through in vitro culture techniques, subsequently infusing these expanded cells into cancer patients as a therapeutic intervention [94]. However, these in vitro-expanded NK cells demonstrate relatively limited persistence in patients following infusion [95]. When cellular telomeres shorten beyond a critical threshold, cells enter a senescent state due to insufficient telomerase activity necessary for proper replication [82, 83]. Studies have demonstrated that premature telomerase inactivation can trigger accelerated senescence through pathways that operate independently of conventional telomere shortening-induced senescence [82]. Enhanced survival and proliferative capacity of NK cells in vitro may paradoxically induce cellular senescence resulting from sustained non-physiological proliferation [26], despite NK cells not typically exhibiting high proliferative potential under normal conditions. In vitro-expanded NK cells exhibit elevated secretion of proinflammatory molecules, including histone proteases and matrix metalloproteinases [96], potentially characteristic of the senescence-associated secretory phenotype (SASP); nevertheless, additional research is necessary to definitively establish whether these phenotypic changes genuinely represent NK cell senescence and to comprehensively assess their long-term implications for immunotherapeutic efficacy.

The function and behavior of NK cells are significantly altered within the tumor microenvironment (TME) and during chronic inflammatory infections. Studies have suggested that abnormal NK cell expansion under these conditions may lead to senescence or functional exhaustion; however, further experimental confirmation remains necessary to substantiate these findings. The TME constitutes a critical factor in tumor growth and progression. It comprises tumor cells, tumor stromal cells (e.g., stromal fibroblasts, endothelial cells), immune cells (including macrophages and lymphocytes), and noncellular components of the extracellular matrix (e.g., collagen, hyaluronic acid) [97]. Furthermore, the TME encompasses various microenvironmental features, including hypoxic, acidic, and innervation ecological niches, as well as metabolic, immune, and mechanical microenvironments. These features collectively influence tumor behavior and therapeutic responses [98]. The chronic inflammatory environment associated with cancer plays a crucial role in tumor formation and progression by enhancing the proliferation and survival of cells harboring oncogenic mutations [99, 100]. In a 2016 study, Aran et al. demonstrated that the epithelial cell-induced inflammatory response observed in a transgenic mouse model was classified as parainflammation [101]. This parainflammatory response has been recognized as a common feature across several human tumors, particularly in bladder, head and neck, cervical, and colorectal cancers [101]. Moreover, inflammatory and immune cells can reprogram the TME through cytokine secretion, facilitate immune cell recruitment, and further promote tumor growth and therapeutic resistance by inducing non-productive immune responses while simultaneously suppressing cytotoxic activity [102]. Therefore, it is plausible that elevated NK cell numbers in the TME may also lead to senescence and, consequently, diminished cytotoxic responses. However, additional research is necessary to translate these findings from in vitro experiments to in vivo models.

## 2. Mechanisms of tumor microenvironment-induced NK cell senescence

### 2.1 TME environment-induced DNA damage response pathway induces NK cell senescence

The activation of CD158d, which induces NK cell senescence, has emerged as a critical area of investigation for understanding the mechanisms underlying NK cell senescence and its physiological implications. The CD158d receptor, also known as KIR2DL4, belongs to the killer cell immunoglobulin-like receptor (KIR) family. KIR family members are essential regulatory receptors predominantly expressed on NK cell surfaces that modulate recognition and cytotoxic responses to target cells through interaction with specific Major Histocompatibility Complex (MHC) molecules. KIR2DL4 represents a functionally distinct KIR receptor that exerts unique regulatory effects on NK cells.

Recent experimental studies have elucidated the mechanism through which the CD158d receptor activates specific and sustained NK cell responses, ultimately inducing a senescent phenotype in human NK cells. This investigation demonstrates that the TRAF6/TAK1 signaling axis activates the CD158d receptor and establishes multiple connections with transforming growth factor beta (TGF-β) and nuclear factor kappa B (NFκB) signaling pathways [14]. Notably, this senescence response is not triggered by conventional mechanisms such as DNA damage or oncogene activation, but rather by the interaction of soluble ligands with endosomal receptors [14] CD158d generates sustained signaling through the DNA-dependent protein kinase catalytic subunit (DNA-PKcs) - protein kinase B (Akt) - NFκB pathway, a characteristic feature of endosomal signaling processes [14]. CD158d activates this pathway via the discoidin domain receptors (DDRs), a subfamily of receptor tyrosine kinases that transmit downstream signals throughout the cascade, consequently inducing the senescence phenotype. Evidence suggests that CD158d-initiated DDR signaling functions as a molecular switch for the senescence-associated secretory phenotype (SASP) in NK cells, potentially promoting vascular permeability and angiogenesis. This mechanism of sustained signaling activation elucidates not only the role of NK cells in maintaining tissue homeostasis but also underscores the beneficial functions of senescent NK cells in maternal vascular remodeling during early pregnancy. This function is critical for reproductive processes, indicating that NK cell senescence represents not merely a natural cellular phenomenon but potentially serves a significant role in systemic physiological regulation. CD158d signaling alone suffices to trigger the DNA damage response and induce a senescent state in NK cells, comparable to that observed in metaphase NK cells. Importantly, FERM domain-containing protein 4B (Frmd4b), a scaffolding protein, exhibits upregulation concomitantly with senescence-associated genes following CD158d-mediated NK cell activation [14, 49].

**Figure 3.**
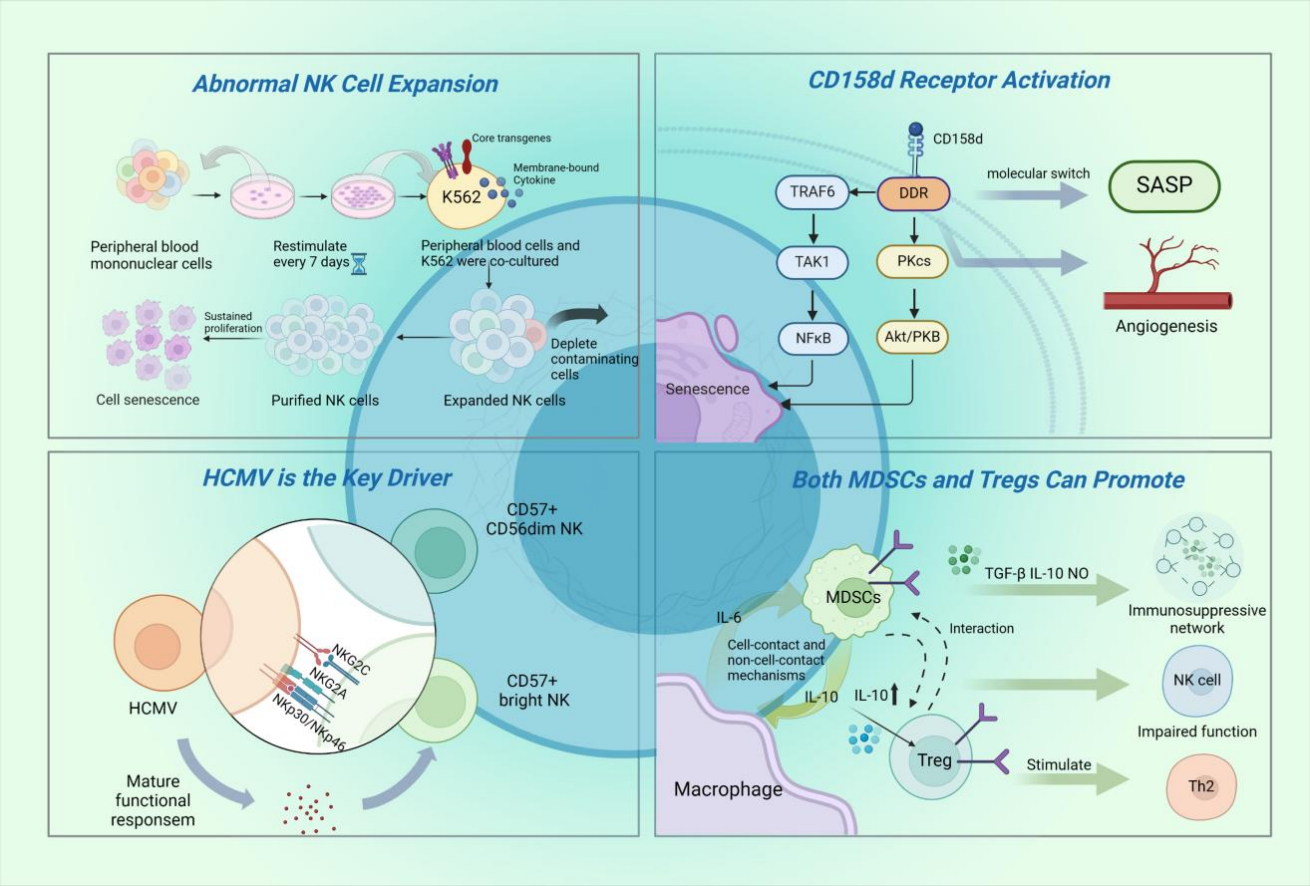
**Mechanism of tumor microenvironment-induced NK cell senescence**. Upper Left: Modified artificial antigen-presenting cells (aAPCs) are commonly used to promote rapid NK cell expansion in vitro to ensure sufficient numbers of NK cells for clinical applications. aAPCs are generated by genetically engineering K562 cells to express co-stimulatory molecules and membrane-bound cytokines. The proliferative activity of NK cells can be maintained through regular stimulation with aAPCs, which can be collected and purified according to clinical requirements, thereby providing an effective cell preparation strategy for NK cell-based immunotherapy. However, these in vitro culture-expanded NK cells are subject to cellular senescence due to insufficient telomerase activity necessary for replication. Upper Right: The CD158d-induced pathway operates through elements of the DDR signaling cascade, thereby generating the NK cell senescence phenotype. Through activation of the TRAF6/TAK1 signaling axis and the DNA-PKcs-Akt-NFκB pathway, the CD158d receptor triggers a series of signaling events. This continuous signaling not only activates the senescence-associated secretory phenotype (SASP), but the activated SASP also exerts significant effects on surrounding tissues, particularly in promoting vascular permeability and neoangiogenesis. Lower Left: Chronic human cytomegalovirus (HCMV) infection is associated with an increased proportion of CD57+CD56dim NK cells and NKG2Cbright NK cells. NKG2Cbright NK cells typically do not express NKG2A and exhibit lower levels of the activating receptors NKp30 and NKp46. Lower Right: Myeloid-derived suppressor cells (MDSCs) block NK cell surface NKp30 and NKG2D receptors to inhibit their function, while regulatory T cells (Tregs) inhibit NK cells via transforming growth factor-β (TGF-β) or the NKG2D complex. Interactions between MDSCs and macrophages in the tumor microenvironment enhance their immunosuppressive capacity. For example, these interactions not only maintain the age-associated immunosuppressive network function and impair NK cell cytotoxicity but also stimulate the T helper 2 (Th2) response. This figure was created using tools provided by Biorender.com (accessed on May 30, 2024). Abbreviations: aAPC, artificial antigen-presenting cells; DDR, Discoidin Domain Receptors; SASP, senescence-associated secretory phenotype; HCMV, human cytomegalovirus; MDSCs, myeloid-derived suppressor cells; Tregs, regulatory T cells.

Sustained activation of CD158d leads to alterations in NK cell morphology and size, which reflect characteristic morphological features of cellular senescence. In particular, CD158d-activated NK cells exhibit a flattened and enlarged morphology, increased SA-β-gal activity [51], and cell cycle arrest in the G0/G1 phase [14]. These features are consistent with the typical manifestations of senescent cells, strongly indicating that CD158d promotes NK cell senescence through the induction of specific signaling pathways. In contrast, IL-2-activated NK cells remain proliferatively active and actively progress through the cell cycle, with the proportion of cells in the G0/G1 phase progressively declining over time. Research has suggested that IL-2 deprivation or exposure to TNF-α leads to apoptosis of NK cells [14]. Furthermore, senescence markers have been found to be highly enriched in metaphase NK cells [103]. To rule out the potential confounding effect of in vitro stimulation on the regulation of these cells, compelling experimental evidence has demonstrated that decidual NK cells are reprogrammed to a senescent state early in gestation. CD158d-stimulated cells and decidual NK cells co-express genes that are critical for the induction and enhancement of the senescent state, including IL-6, IL-8, IL-1β, and p21. p21 (CDKN1A), a major determinant of senescence, was found to be highly expressed during the studied pregnancy period (6-12 weeks). In addition to morphological changes, CD158d-activated NK cells demonstrate prolonged survival without entering the cell cycle, a characteristic that reflects the resistance of senescent cells to apoptosis [14]. Morphological and functional changes in senescent NK cells, as well as their role in vascular remodeling and angiogenesis, emphasize the importance of in-depth studies of NK cell senescence mechanisms for understanding immune system function and potential therapeutic applications. A detailed schematic of the mechanism of NK cell senescence is shown in Figure 3. These findings provide new research directions for studying cancer development associated with NK cell senescence.

The DNA damage response (DDR) is a complex signal transduction pathway that detects DNA damage and replication stress, and subsequently initiates a series of finely regulated reactions to maintain cellular genomic integrity [104-107]. The core of the DDR pathway is mediated by members of the phosphatidylinositol 3-kinase-related protein kinase (PIKKs) family, including ATM, ATR, and DNA-PK, along with members of the poly(ADP-ribose) polymerase (PARP) family [108]. Upon activation, ATM and ATR amplify DDR signals by phosphorylating downstream substrates, such as mediator proteins [109]. These kinases coordinate various cellular activities, including DNA replication, repair, transcription, metabolism, and RNA splicing [110]. A key downstream target in this pathway is p53, which is regulated by ATM and CHK2, leading to the induction of cell cycle arrest, apoptosis, or senescence [109]. Importantly, p53 undergoes negative feedback regulation by WIP1 phosphatase and MDM2 E3 ubiquitin ligase, creating a cyclical activation-inhibition loop [111]. p53 also plays a critical role in regulating cellular senescence, as studies have demonstrated that p53 deficiency can rescue some senescent phenotypes while simultaneously increasing cancer risk [112].

DDR serves as a central mediator of cellular senescence, exhibiting distinct mechanisms and functions in normal aging compared to tumor microenvironment (TME)-induced senescence, although the underlying mechanisms of these differences still require further experimental investigation. In the normal aging process, DDR is primarily driven by endogenous factors, including telomere shortening, genomic instability, and accumulated oxidative damage [11, 83]. During this process, key molecules including ATM/ATR kinases, CHK1/CHK2 kinases, and p53 orchestrate crucial regulatory functions, collectively coordinating cellular senescence [108]. In TME-induced senescence, DDR activation occurs in response to a diverse array of exogenous factors. Hypoxia, nutrient deprivation, metabolic waste, and immunosuppressive factors secreted by tumor cells in the TME collectively trigger DNA damage and subsequent DDR activation in NK cells. For example, the hypoxic environment in the TME stimulates the production of reactive oxygen species (ROS), resulting in DNA oxidative damage and subsequent activation of the DDR pathway [26]. Additionally, immunosuppressive factors secreted by tumor cells, notably TGF-β and IL-10, may promote DNA damage accumulation and DDR activation by suppressing DNA repair mechanisms [15].

### 2.2 Sustained tumor antigen stimulation may be a key factor driving immune senescence of NK cells

Sustained tumor antigenic stimulation represents a crucial factor to be considered when investigating NK cell dysfunction in the tumor immune microenvironment. Chronic human cytomegalovirus (HCMV) infection promotes immune senescence and attenuates NK cell immune responses by elevating the proportion of CD57+ CD56dim NK cells [22]. During such infections, the virus persists in the host for extended periods, and the host's immune system fails to completely eradicate it, resulting in ongoing viral replication. Chronic human cytomegalovirus (CMV) infection is strongly associated with the deterioration of adaptive and innate immune functions and is considered a key factor in the immune system aging process; these infections are widely acknowledged as the primary driver of immune senescence [113, 114]. Substantial evidence establishes HCMV as a significant contributor to the diminished functionality of T cells and NK cells in immune senescence [115]. HCMV seropositivity is associated with an increased prevalence of CD57+ CD56dim NK cells, a phenomenon that is predominantly observed in HCMV-infected individuals. Furthermore, HCMV infection promotes the maturation of lymphocyte subsets characterized by CD57 expression and enhances their functional responses to stimuli. Notably, HCMV-infected young adults exhibit a similar proportion of CD57+ NK cells as HCMV-carrying older adults, both being significantly higher than those observed in HCMV-uninfected young adults [113]. Recent research has linked HCMV to a sustained increase in NKG2C bright NK cells in healthy adults and children. Characteristically, these cells do not co-express NKG2A and exhibit lower levels of the activation receptors NKp30 and NKp46 [115].

Sustained tumor antigen stimulation has been identified as a key factor driving immune senescence, also commonly referred to as immune depletion, particularly in the context of cancer and chronic infections. Prolonged exposure to high levels of antigens, such as tumor antigens, leads to T-cell hypoplasia, characterized by decreased proliferative capacity, impaired cytotoxic function, and reduced ability to secrete essential cytokines like IFN-γ [116]. Similarly, in the tumor microenvironment (TME), persistent tumor antigen stimulation frequently leads to NK cell exhaustion, which impairs their immune surveillance function and contributes to tumor immune escape [34]. The primary characteristics of NK cell exhaustion include impaired cytotoxic function, reduced cytokine secretion, upregulation of inhibitory receptor expression, down-regulation of activating receptor expression, dysregulated proliferation, and metabolic dysfunction [117]. While both NK cell immunosenescence and exhaustion represent states of functional impairment, they fundamentally differ in their underlying mechanisms and biological nature [26]. Both phenomena can manifest as functional impairments such as reduced proliferative capacity [118], decreased cytotoxicity, and diminished cytokine secretion [119], and both may occur under chronic stimulation conditions commonly observed in tumors [120]. However, senescence is primarily driven by intrinsic factors or chronic inflammation, characterized by cell cycle arrest and relatively irreversible functional loss, whereas exhaustion results from persistent antigen stimulation [121-123], is distinguished by high expression of inhibitory receptors [124], and may achieve partial functional recovery through blockade of relevant pathways [125-128]. Therefore, distinguishing between these concepts enables more precise understanding of NK cell dysfunction and facilitates the development of more effective immunotherapeutic strategies [129, 130]. A comprehensive comparison of NK cell immune-senescence and exhaustion characteristics is presented in Table 1. Sustained tumor antigen stimulation may serve as a driver of NK cell immune senescence, as it results in persistent activation and subsequent depletion of NK cell function, leading to a decline in their cytotoxic efficacy. It remains unclear whether NK cell senescence and depletion represent separate and distinct functional abnormalities, despite both typically being characterized by a decline in effector function or proliferation [11]. Understanding these processes is crucial for the development of novel immunotherapies and ultimately improving patient prognosis.

**Table 1:** Systematic comparison of NK cell senescence and failure.

Feature	NK Cell Senescence	NK Cell Exhaustion
**Core Definition**	Irreversible impairment of effector functions driven by intrinsic cellular factors or chronic, persistent inflammation	Reversible dysfunction of effector functions induced by chronic antigen stimulation
**Driving Factors**	Accumulation of DNA damage, telomere shortening, mitochondrial dysfunction, epigenetic modifications, chronic inflammation, etc.	Sustained antigen stimulation, absence or insufficiency of cytokines (e.g., IL-12, IL-15), activation of immune checkpoint pathways (e.g., PD-1, TIM-3), etc.
**Cell Cycle Status**	Cell cycle arrest, G0/G1 phase blockade, impaired proliferative capacity	Cell cycle arrest, impaired proliferative capacity, but generally retains more proliferative potential than senescent cells
**Functional Features**	- Significantly reduced cytotoxicity - There was a marked decrease in proliferative capacity - Decreased cytokine (IFN-γ, TNF-α) production - Decreased proportion of mature subsets - Metabolic alterations (reduced mitochondrial function) - Secretion of SASP factors	- Decreased cytotoxicity - Decreased proliferative capacity - Multiple cytokine secretion disorders - Memory-like deficits - Impaired responses to activation signals
**Key Molecular Markers**	↑ p16INK4a, p21CIP1, SA-β-gal,CD57, CD27,KIRs,NKG2A ↓ NKG2D, NKp30	↑ PD-1, TIM-3, LAG-3, CD39, CD96,TIGIT,KIR3DL2,NKG2A ↓ NKG2D, NKp30,NKp46,NKp44,NKp80,DNAM-1, CD16
**Molecular Mechanisms**	- DNA damage response Activation (DDR) - Impairment of mitochondrial oxidative phosphorylation - A compensatory increase in glycolysis was observed - SASP factor release	- The expression of cytotoxic granules (perforin, granzyme B) was decreased - Reprogramming of lipid metabolism - Hyperbreakdown of glutamine
**Reversibility**	Irreversible (senescence-associated secretory phenotype, SASP persistence)	Partially reversible (PD-1/IL-2 blockade may restore function)
**Microenvironment**	Inflammatory factors (e.g., TNF-α, IL-6) accelerate NK cell senescence. Factors secreted by tumor cells or stromal cells may induce NK cell senescence.	Immunosuppressive cells (e.g., MDSCs, TAMs) and cytokines (e.g., TGF-β, IL-10) within the tumor microenvironment promote NK cell exhaustion. Hypoxia and accumulation of metabolites (e.g., adenosine) also contribute.
**Clinical Associations**	Cancer (tumor microenvironment promotes NK cell senescence), chronic inflammation, age-related diseases (e.g., cardiovascular disease, neurodegenerative disorders)	Chronic viral infections (e.g., HIV, CMV), cancer, autoimmune diseases
**Intervention Strategies**	mTOR inhibitors	PD-1/PD-L1 blocking agents, TIM-3 inhibitors, CAR-NK cell therapy

Abbreviations: mTOR, Mammalian Target of Rapamycin; SASP, Senescence-Associated Secretory Phenotype; DDR, DNA Damage Response; DNAM-1, DNAX Accessory Molecule-1; TAM, Tumor-Associated Macrophage; TIM-3, T-cell Immunoglobulin and Mucin-domain Containing-3; LAG-3,Lymphocyte-Activation Gene 3; TIGIT, T cell Immunoreceptor with Ig and ITIM domains.

Although persistent antigen stimulation is widely considered a critical driving factor for NK cell senescence, our current understanding of its detailed molecular mechanisms remains relatively limited. Existing literature primarily focuses on NK cell exhaustion in chronic viral infections and tumor microenvironments, while providing insufficient evidence that directly examines how persistent antigen stimulation specifically induces NK cell senescence. Therefore, further in-depth research is needed to elucidate the relevant signaling pathways, epigenetic alterations, and metabolic reprogramming necessary to comprehensively understand the specific role of persistent antigen stimulation in NK cell senescence.

### 2.3 Both MDSCs and Tregs in TME promote NK cell senescence

Both MDSCs and Tregs play crucial roles in the immune system, particularly in inducing immune senescence of NK cells. MDSC-induced immune senescence encompasses several aspects, including alterations in NK cell subsets, reduced cytotoxicity, decreased cytokine production, and diminished response to cytokines [40]. MDSCs inhibit NK cell function by blocking NKp30 and NKG2D receptors on NK cell surface, while Tregs further suppress NK cells via TGF-β or NKG2D complexes [131]. Research has demonstrated that TGF-β, IL-10, and nitric oxide (NO) are crucial soluble mediators secreted by MDSCs that maintain age-related immunosuppressive network function [40]. In the cancer context, elevated levels of TGF-β significantly impair NK cell function by converting these cells into less efficient phenotypes, consequently compromising NK cell function through various immune evasion mechanisms. Thus, strategies targeting TGF-β inhibition may represent an effective approach to enhance NK cell immune function [132]. Furthermore, interactions between MDSCs and macrophages, through both cell-contact and non-cell-contact mechanisms, enhance the immunosuppressive capacity of these cells, as observed in the TME. These interactions not only increase IL-10 production and activate immunosuppressive Tregs but also stimulate Th2 responses while decreasing antigen presentation and impairing the cytotoxicity of CD8+ and NK cells [133-135]. In summary, MDSCs and Tregs contribute to immunosuppression within the immune system and NK cell immune senescence through a series of complex interactions and signaling pathways, particularly through the inhibition of NK cell function, ultimately impairing the immune system's ability to respond to tumors and pathogens.

### 2.4 Inhibitory cytokines in TME induced NK cell senescence

The chronic inflammatory state within tissues also initiates numerous immunosuppressive responses that function to counteract the deleterious effects of persistent inflammation [92, 93, 136]. In the TME, suppressor cytokines including transforming growth factor-β (TGF-β) and IL-6 play a crucial role in promoting immune senescence by modulating the function of NK cells. Specific immunosuppressive cell types, such as myeloid-derived suppressor cells (MDSCs), regulatory T cells (Tregs), and M2-type macrophages, contribute to immune senescence by secreting a diverse array of immunosuppressive molecules, including TGF-β, IL-10, reactive oxygen species (ROS), arginase-1 (ARG1), and indoleamine 2,3-dioxygenase (IDO). These molecules impair the function of a wide spectrum of immune cells, including CD4+ and CD8+ T cells, B cells, macrophages, NK cells, and dendritic cells, thereby establishing an immunosuppressive network [40, 137, 138]. Furthermore, mesenchymal stem cells (MSCs) initially promote NK cell function by releasing type I interferon; however, they subsequently induce NK cell dysfunction by secreting TGF-β and IL-6, which ultimately leads to NK cell senescence [34]. A study by the authors revealed a time-dependent regulatory mechanism, whereby activated MSCs in the initial phase enhance NK cell function by secreting type I interferon [139]. However, over time, this stimulatory effect is replaced by the inhibitory actions of TGF-β and IL-6, which restrict the effector function of NK cells and terminate the inflammatory response by promoting the transition of NK cells to a regulated senescent phenotype [139].

## 3. The role of senescent NK cells in tumor immunity

Overall, NK cell senescence significantly impairs their ability to eliminate tumor cells and substantially contributes to tumor progression.

### 3.1 NK cell senescence status can be used as a marker for monitoring antitumor efficacy

The precise relationship between NK cell senescence status and antitumor efficacy remains to be fully elucidated. Nevertheless, the role of NK cells in tumor immunology represents an active and rapidly evolving area of investigation. Numerous studies are currently investigating the therapeutic potential of NK cells, including their cytolytic activity, regulatory mechanisms, and innovative strategies to enhance antitumor responses through functional augmentation. NK cells play a fundamental role in human immune surveillance and are primarily responsible for recognizing and eliminating aberrant cells, particularly tumor cells [49, 56, 64, 73, 140]. The functional capacity of NK cells progressively declines with age or in specific disease states, and this senescent phenotype significantly compromises their antitumor efficacy. Utilizing NK cell senescence status as a clinical biomarker presents multiple methodological challenges, including the precise definition and reliable quantification of NK cell senescence, as well as establishing robust correlations between these measurements and clinical outcomes. Future research must systematically address these challenges to effectively harness the therapeutic potential of NK cells in antitumor interventions.

**Figure 4.**
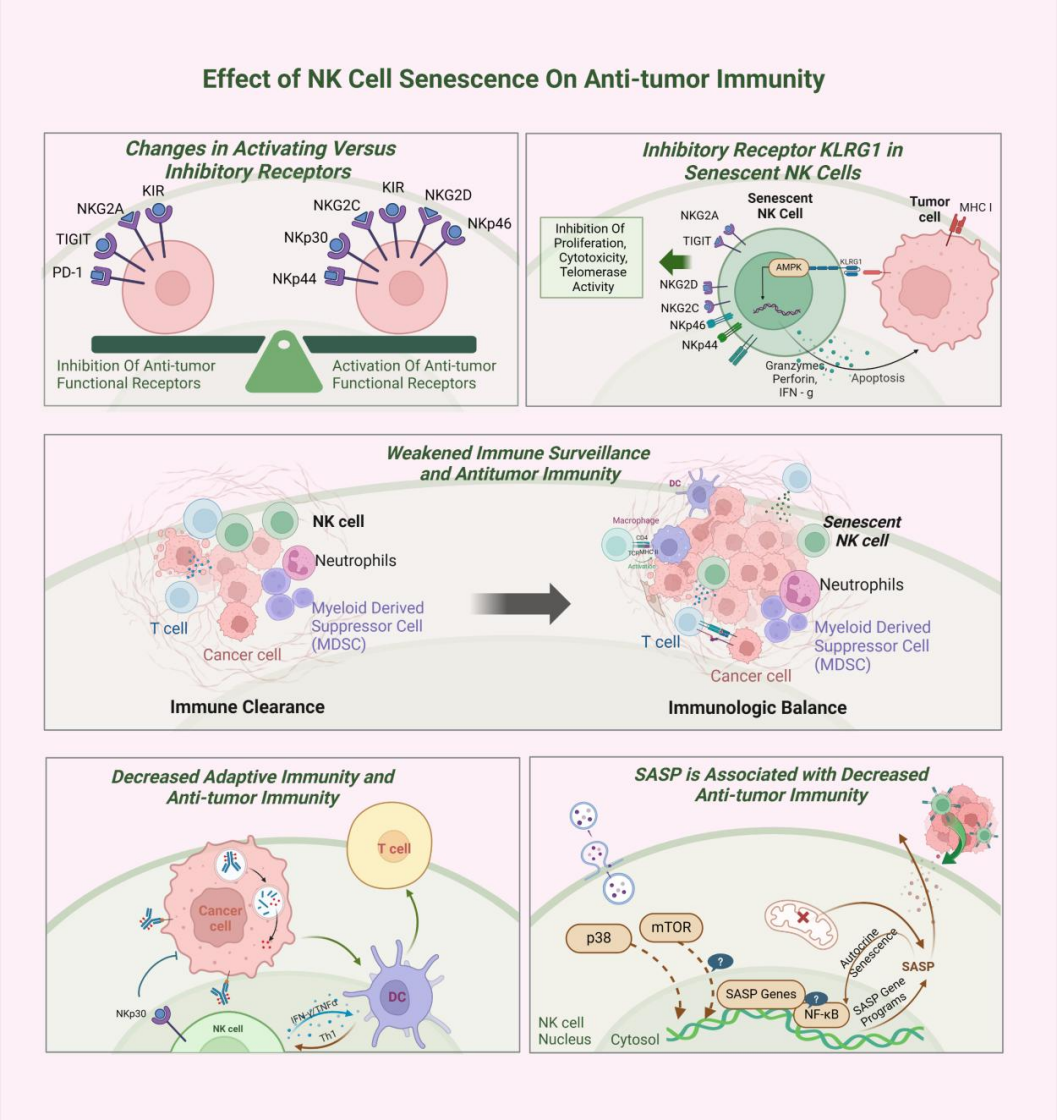
**Effects of NK cell senescence on antitumor immunity**. Upper Left: The scale in the figure illustrates how the anti-tumor function of NK cells is coordinated and counterbalanced by the equilibrium between inhibitory and activating receptors on the cell surface. The figure also categorizes and enumerates the most prevalent and crucial inhibitory and activating receptors expressed on NK cells. Upper Right: This panel demonstrates how KLRG1 stimulates AMPK activity in NK cells with high KLRG1 expression, subsequently inhibits telomerase activity, suppresses NK cell proliferation and cytotoxicity through this signaling pathway, and reduces IFN-γ production. Middle Panel: The role of senescent NK cells in immune surveillance is diminished, while their reduced aggressiveness attenuates the development of chronic sterile inflammation, thereby maintaining tissue homeostasis and immune balance. Lower Left: This panel illustrates the cellular interactions underlying both declining adaptive immunity and declining anti-tumor immunity. Recognition of specific ligands on DCs by NKp30 regulates NK cell-DC interactions, promotes the release of IFN-γ and TNF-α, and triggers DC maturation. Tumor- or viral antigen-activated DCs effectively activate T cells, while Th1-type cytokines released by these activated DCs further enhance the activation status of NK cells. Decreased interaction between senescent NK cells and DCs results in less effective adaptive immune responses to viral infections and malignant cells. Lower Right: SASP signaling mediated by NF-κB, mTOR, and p38 MAPK creates a favorable cytokine microenvironment for tumorigenesis. Decreased mitochondrial autophagy leads to increased mitochondrial content and defects in the mitochondrial network, resulting in SASP development and metabolic dysfunction during cellular senescence. This figure was created using tools provided by Biorender.com (accessed on May 30, 2024). DC, dendritic cell; mTOR, mammalian target of rapamycin; p38 MAPK, p38 mitogen-activated protein kinase.

The senescent phenotype of NK cells is intricately associated with diverse clinical pathophysiological processes; consequently, employing NK cell senescence markers for patient stratification, auxiliary diagnostic approaches, and prediction of treatment responses holds substantial translational significance. This rapidly emerging field represents a pivotal focus for future experimental and translational research and will likely make substantial contributions to the diagnosis, prognosis, and treatment optimization of cancer patients in clinical settings. NK cell senescence markers exhibit considerable clinical utility in patient stratification, complementary diagnostic approaches, and accurate prediction of treatment responses. Patient stratification based on senescence markers (including CD57, KIR, and NKG2A) effectively identifies individuals with variable disease risks and distinct prognostic outcomes; specifically, an elevated proportion of CD57+ NK cells in cancer patients consistently correlates with poor clinical prognosis [39]. Senescence markers demonstrate significant potential in facilitating the diagnosis of chronic infections and autoimmune conditions, comprehensively assessing immune functional status, and informing evidence-based clinical decisions, as exemplified by the established correlation between NK cell senescence profiles and viral control efficacy in HIV infection [141]. Furthermore, senescence markers serve as robust predictors of responses to tumor immunotherapy; notably, the degree of NK cell senescence prior to immune checkpoint inhibitor administration strongly correlates with therapeutic efficacy, and selective utilization of non-senescent, functionally competent NK cells for adoptive cell therapy significantly enhances treatment success rates. Therefore, systematic and comprehensive investigation of NK cell senescence markers holds substantial translational potential for diverse clinical applications and personalized therapeutic approaches.

### 3.2 NK cell senescence suppresses tumor immunity

Within the oncology field, there is currently insufficient direct evidence demonstrating that NK cell senescence directly causes a decline in anti-tumor immunity. However, considering the critical role of NK cells in anti-tumor immunity, it is reasonable to hypothesize that NK cell senescence significantly impacts anti-tumor immune responses. Therefore, elucidating the relationship between established characteristics of NK cell senescence and diminished anti-tumor immunity represents a critical avenue for future research. The impact of NK cell senescence on anti-tumor immunity is illustrated in Figure 4.

### 3.2.1 Changes in activating and inhibitory receptors

The anti-tumor function of NK cells is regulated through a delicate balance between inhibitory and activating cell surface receptors [26, 72, 94]. Additional alterations in the expression of activating and inhibitory receptors have been observed in NK cells isolated from tumor patients, potentially resulting from their interaction with tumor cells [35]. Functional deterioration of NK cells, which constitute the first line of defense against viral infections and malignant cells, is believed to partially account for the increased cancer incidence in older adults [72]. Functionally intact NK cells play a significant role in tumor surveillance and elimination. Classical inhibitory receptors include the killer immunoglobulin-like receptors (KIR) and the C-type lectin family member CD94/NKG2A, which recognize self MHC class I molecules [4]. While the CD94-NKG2A signaling pathway remains unchanged, senescence induces an increase in killer cell immunoglobulin-like receptor (KIR) expression, a decrease in CD94-NKG2A expression, and reduced expression of activation receptors (e.g., NKp30 and NKp46) in aged NK cells [26, 142]. The production efficiency of IFN-γ decreases, as does the ability of IFN-α and IFN-β to enhance NK cytotoxicity [70], consequently diminishing the immune effects on the tumor microenvironment (TME). Sustained expression of NKG2D is considered essential for maintaining NK cell function [26]. Alterations in classical major MHC-I specific inhibitory receptors, including changes in NK cell subpopulations and receptor expression patterns, play a critical role in cancer pathogenesis [26]. T-cell immunoglobulin and immunoreceptor tyrosine-based inhibitory motif (ITIM) domain (TIGIT) are well-established checkpoint receptors that modulate the antitumor activity of NK cells [143]. However, our current understanding of the underlying mechanisms governing these impairments remains limited and incomplete.

The inhibitory receptor KLRG1, when highly expressed, is thought to exert its negative regulatory function in highly differentiated and senescent NK cells through the activation of adenosine monophosphate-activated protein kinase (AMPK) [12]. KLRG1 has been documented to be highly expressed in NK cells infiltrating various solid tumors and leukemias, including breast cancer and myelodysplastic syndromes (MDS) [144, 145]. This signaling pathway likely represents one of the mechanisms by which NK cell senescence leads to diminished anti-tumor function. AMPK, an intracellular signaling sensor, integrates signals from low ATP levels and DNA damage to orchestrate cellular functions [12]. In KLRG1-overexpressing NK cells (KLRG1bright), KLRG1 has been shown to stimulate AMPK activity independently of its classical inhibitory target Akt. This AMPK activation subsequently suppresses telomerase activity, proliferation, cytotoxicity, and IFN-γ production in NK cells through downstream signaling events [146, 147]. The interaction between KLRG1 and AMPK highlights the central role of energy metabolism in NK cell senescence and subsequent impairment of antitumor function. Despite these findings, the role of KLRG1 in senescence remains unclear, and the precise mechanism by which KLRG1 induces inhibition of NK cell function is not fully elucidated [12]. Upon activation, AMPK triggers p38 MAPK phosphorylation, and pharmacological inhibition of either molecule has been demonstrated to restore proliferation and telomerase activity [12]. Collectively, these observations suggest that inhibition of KLRG1/AMPK signaling could potentially restore NK cell function, thereby leading to immune enhancement in both aging-related senescence and cancer patients.

### 3.2.2 SASP and anti-tumor immune decline

The SASP secretome from senescent cells exerts a profound impact on the surrounding tissue microenvironment. SASP comprises a variety of factors, including pro-inflammatory cytokines, chemokines, growth factors, ROS, angiogenic factors, and proteases, which collectively create a favorable cytokine microenvironment for tumorigenesis [26]. SASP is regulated by several key signaling pathways, including NF-κB, mammalian target of rapamycin (mTOR) [148], and p38 mitogen-activated protein kinase (p38 MAPK) [142], all of which are associated with reduced mitochondrial autophagy. Notably, reduced mitochondrial autophagy results in increased mitochondrial mass and defects in the mitochondrial network, potentially contributing to SASP development and metabolic dysfunction during senescence [26]. While these pathways have been well-documented in experimental studies of T-cell senescence, p38 MAPK has only shown preliminary indications of its role in anti-tumor immunity in NK-cell senescence, highlighting significant potential for further research in NK cells. Importantly, SASP exhibits a dual role: on one hand, it may promote tumor growth, while on the other hand, it may recruit immune cells, particularly NK cells, to facilitate the clearance of senescent cells, thereby preventing tumorigenesis [149]. Therefore, senescent cells and their associated SASP components play complex and crucial roles in both carcinogenesis and immune response regulation. Admittedly, this hypothesis remains at a preliminary stage and requires rigorous experimental validation to definitively establish its relevance in NK cell biology.

### 3.2.3 Decreased adaptive immunity and decreased anti-tumor immunity

NK cell senescence may significantly impair the crosstalk between the innate and adaptive immune systems [50]. The cytotoxic function of NK cells is intricately associated with the expression of their cell surface receptors. In older adults, the expression of NKp30, a critical activating receptor of NK cells, is significantly reduced, thereby compromising the interaction between NK cells and dendritic cells (DCs). The NKp30 receptor mediates the interaction between NK cells and DCs through recognition of specific ligands expressed on DCs. This interaction can either result in direct killing of DCs by NK cells or induce the release of IFN-γ and tumor necrosis factor-α (TNF-α), which subsequently triggers DC maturation. DCs that have been activated by tumor or viral antigens demonstrate enhanced capacity to activate T cells with greater efficiency. Reciprocally, T helper 1 (Th1)-type cytokines released by activated DCs further augment the activation status of NK cells [85]. As individuals age, NK cell function undergoes significant decline, characterized predominantly by decreased NKp30 receptor expression and attenuated granule-mediated cytotoxicity. These alterations not only directly compromise the ability of NK cells to mount an immediate response to transformed cells but also diminish the efficiency of the adaptive immune response [4]. The diminished interaction between senescent NK cells and DCs leads to an impaired adaptive immune response against viral infections and malignant cells. The downregulation of NKp30 on elderly-derived NK cells further compromises the interactions between these immune cells, consequently limiting their collaborative capacity to initiate adaptive immune responses against viral infections and tumor cells [3].

### 3.2.4 Complexity of diminished immune surveillance and antitumor immunity

Cellular senescence establishes a favorable microenvironment through the SASP mechanism, which protects tumor cells from immune clearance [150]. Within the context of the senescent immune system, T cells play a crucial role in cancer immunosurveillance. With advancing age, both the number and frequency of naïve T cells in the CD4+ and CD8+ subsets significantly decrease. NK cells play a vital role in complementing adaptive T cell-mediated, MHC-restricted immune responses, especially when targeting MHC class I-negative cells. By compensating for this limitation of T cells, NK cells contribute to the elimination of aberrant cells, including human leukocyte antigen (HLA) class I-negative tumor escape variants, thus providing durable immune protection. Although the hypothesis that senescent NK cells have diminished capacity in immune surveillance seems intuitive, experimental confirmation is still needed, as it remains an open question whether immunosenescent NK cells accumulate with age [26]. Notably, immunosurveillance may also imply a reduced attack on normal cells, potentially mitigating tissue damage. This attenuated aggressiveness may contribute to the prevention of chronic sterile inflammation, ultimately facilitating the maintenance of tissue homeostasis.

## 4. Ways to delay NK cell senescence

### 4.1 Delaying NK cell senescence by adding cytokines

When exploring the potential of NK cell immunotherapy, researchers have investigated the effects of cytokines such as IL-2, IL-15, and IL-21 on NK cell telomerase activity, telomere length, and their role in delaying NK cell senescence. IL-2, IL-15, and IL-21 are all members of the common γ-chain receptor family, and their effects on NK cells have been well documented [11, 37]. Studies have demonstrated that these cytokines significantly enhance NK cell immune function, prolong their lifespan, and delay senescence. Under laboratory conditions, cell growth can be significantly manipulated; however, normal, healthy human NK cells cultured in vitro exhibit a growth limit of approximately 15 weeks [90]. In mouse experiments, P53-deleted mouse NK cells could be cultured in vitro for more than a year under specific conditions, whereas the proliferative capacity of normal, healthy NK cells in vitro is usually limited to 7-10 days, after which they begin to exhibit changes associated with apoptosis [90]. The research model overcame this proliferation limit by using transgenic K562 cell lines that express membrane-bound forms of 4-1BBL and IL-15 and culturing healthy human NK cells in the presence of 10 IU/mL of IL-2 [11].

### 4.1.1 IL-21 significantly delays NK cell senescence

Among these cytokines, IL-21 demonstrated superior efficacy in increasing NK cell telomere length, particularly through the STAT3 component of the JAK/STAT signaling pathway [29, 37, 77, 151]. IL-21-expanded NK cells preserved the donor KIR repertoire in terms of phenotype and cytotoxic activity, exhibiting elevated expression levels of natural cytotoxicity receptors (NCRs), CD16, and NKG2D. Concurrently, IL-21-expanded NK cells displayed increased transcript levels of the activation receptor CD160, while maintaining comparable mRNA expression of 96 other genes, suggesting robust cytokine secretion capacity and potent cytotoxicity against various tumor cell lines [37]. Therefore, IL-21-expressing artificial APCs actively promote human NK cell proliferation, resulting in increased telomere length and reduced senescence markers [26]. The findings support the clinical potential of this approach for NK cell-based adoptive immunotherapy [37]. Nevertheless, the direct impact of IL-21 and STAT3 activation on NK cells remains both elusive and debatable.

### 4.1.2 Effects of other cytokines on NK cell senescence

Comprehensive studies have demonstrated that IL-2 significantly enhances telomerase activity in NK-92 cells at both the transcriptional and post-translational levels via activation of the PI3K/Akt signaling pathway [151]. Furthermore, the translation process of IL-2 in NK cells is regulated by Akt, Hsp90, mTOR, and S6K complexes, suggesting that the mechanism of telomerase regulation in NK cells differs from that in other cell types [37, 151]. The uniqueness of this regulatory mechanism in NK cells offers a new perspective on the role of telomerase in immunomodulation. Importantly, the observation that Hsp90 and mTOR are involved in human telomerase activity in primary cells from patients with NK cell lymphoma or leukemia corroborates the upregulation of the Akt/Hsp90/mTOR/S6K signaling pathway in telomerase activity observed in these patients [151]. In contrast to IL-15, which transmits its signal primarily through the activation of STAT5 [37, 152], the involvement of STAT5 in IL-21 signaling is significantly more limited. In the field of NK cell research, in addition to IL-2, the effects of IL-15 on both telomerase activity and proliferation of NK cells have garnered substantial attention. However, although the effects of IL-15 and IL-7 on the telomerase activity of CD8+ cells have been well documented, their specific effects on NK cells remain elucidated. This gap in knowledge, in contrast to the known stimulatory effects of IL-2 and IL-21, underscores the need for further research [29]. Collectively, these four cytokines enhance immune cell lifespan through similar mechanisms, primarily by stimulating telomerase activity or by inhibiting apoptosis [29]. Therefore, an in-depth exploration of the role of these cytokines in regulating telomere length in NK cells is not only crucial for understanding the biological functions of NK cells but may also yield novel therapeutic strategies for NK cell-related diseases.

### 4.2 Restoration of senescence after NK cell expansion by modifying the key gene

Human telomerase reverse transcriptase (hTERT) is a key gene targeted by cytokines that regulates senescence in NK cells; therefore, hTERT gene modification can restore NK cell function after expansion-induced senescence. IL-2 enhances NK cell proliferation by upregulating hTERT mRNA levels and telomerase activity [151]. Similarly, IL-15 primarily signals through STAT5, and hTERT gene modification can restore IL-15-mediated senescence following NK cell expansion, albeit with increased cytogenetic instability. Additionally, the STAT3 component of the IL-21 signaling pathway activates hTERT [37, 90, 152]. Interestingly, NK cells expanded with IL-15 exhibit shorter telomere lengths compared to those expanded with soluble IL-2; however, this phenomenon may be attributed to enhanced NK cell proliferation rather than diminished hTERT activity. Moreover, elevated hTERT expression may promote cell survival through telomere length-independent mechanisms [153]. In contrast, NK cells expanded with IL-21 displayed longer telomere lengths compared to those expanded with IL-15, emphasizing the distinct role of IL-21 in enhancing NK cell function and inhibiting senescence. In fact, TERT overexpression enables sustained growth and maintenance of NK cell function far beyond the senescence-induced turning point [90]. These comprehensive studies not only enhance our understanding of the mechanisms underlying NK cell senescence and immunoregulation but also lay the foundation for developing novel immunotherapeutic approaches based on hTERT gene modification for NK cells. These findings underscore the potential value of cytokines and gene modifications in augmenting the therapeutic efficacy of NK cells, particularly in cancer immunotherapy, by improving NK cell persistence and function.

Recent studies have revealed the critical roles of PI3K/Akt and mTOR signaling pathways in regulating hTERT activity in NK cells through experiments utilizing specific reagents. Specifically, LY294002, a PI3K-specific inhibitor, effectively blocked PI3K-dependent Akt activation, consequently inhibiting hTERT mRNA expression and telomerase activity in NK cells. This finding emphasizes the crucial role of the PI3K/Akt pathway in regulating telomerase activity. Furthermore, rapamycin, an mTOR pathway inhibitor, blocked the enhancement of telomerase activity in IL-2-stimulated NK-92 cells, despite not affecting hTERT mRNA or protein expression [151]. Moreover, rhizoglobin and rapamycin were found to block IL-2-stimulated translocation of hTERT from the cytoplasm to the nucleus, which provides further support for the notion that Hsp90 and mTOR regulate IL-2-induced telomerase activity at the post-translational level [151]. These findings offer novel insights into the molecular mechanisms underlying NK cell function regulation and provide potential targets for developing anticancer strategies that leverage NK cell-mediated immunity.

### 4.3 Delayed NK cell senescence increases CAR-NK cell efficacy

CAR-NK cell therapies offer several unique advantages over CAR-T cells, including a more favorable safety profile, greater accessibility, streamlined preparation, and effective tumor targeting capabilities. These distinctive properties position NK cells as a promising candidate for off-the-shelf immune cell therapy [28]. Cytokine release syndrome (CRS) and neurotoxicity, common side effects associated with CAR-T cell therapy, occur significantly less frequently in CAR-NK cell therapy, establishing it as a safer alternative [28, 36, 87, 154, 155]. Moreover, the procurement and preparation of NK cells are comparatively straightforward, as they can be isolated from various sources, including peripheral blood from patients or healthy donors. This broad range of sources substantially reduces production costs and may accelerate the clinical adoption of CAR-NK cell therapy [28]. Furthermore, unlike T cells, NK cells can be preactivated without prior licensing through exposure to IL-2 or IL-15 [28]. In contrast to CAR-T cells, which require weeks for customization and production, CAR-NK cells can significantly expedite the treatment process as an "off-the-shelf" therapeutic option. Recent clinical trial data highlight the potential of CAR-NK cell therapy across a broad spectrum of tumor types while underscoring the necessity to further optimize CAR structures and enhance NK cell persistence [28]. However, CAR-NK cell therapy still faces several challenges, including in vitro expansion and activation of primary NK cells, complications in the storage and transportation of NK cell products, off-target effects, immune-related toxicities, and practical implementation in clinical settings. Consequently, further research is warranted to optimize the efficacy and application of CAR-NK cell therapy [28]. Through meticulous engineering of NK cells, future studies are expected to further enhance their therapeutic efficacy and safety, opening new avenues in cancer therapy.

Currently, research and development of novel NK cell therapies for cancer treatment focuses on three key areas: NK cell activation and expansion (see above), combination therapies utilizing NK cells with immune checkpoint inhibitors [86, 156], and genetically engineered NK cell therapy [28, 157]. Among these promising approaches, genetically engineered NK cell therapy involves genetically modifying NK cells to express specific chimeric antigen receptors (CARs) using advanced gene editing techniques such as CRISPR-Cas9 [36]. These CAR-NK cells can specifically recognize and attack tumor cells expressing the corresponding antigens, exhibiting high efficiency in targeting and eliminating malignant cells while producing relatively low side effects. This approach offers promising potential for the treatment of certain refractory cancers [36].

However, before advancing these approaches to clinical applications, we must systematically acknowledge and investigate their inherent limitations. Adoptive NK cell therapy aims to enhance anti-tumor activity through ex vivo expansion and activation of NK cells; however, in vitro manipulation may accelerate NK cell senescence processes, potentially compromising therapeutic outcomes [35]. Cytokine therapy, while capable of promoting NK cell proliferation and functional recovery, may induce immune-related toxicities, especially in elderly patients [11]. Immune checkpoint inhibitors, such as PD-1 antibodies, can alleviate NK cell inhibition; nevertheless, not all patients benefit from these agents, and they may exacerbate autoimmune responses [86]. Finally, the feasibility of clinical translation remains fundamental to the success of the aforementioned strategies. The complexity of gene editing techniques, high production costs, and stringent regulatory requirements collectively limit their widespread clinical implementation. Similarly, cytokine therapy faces significant challenges regarding administration protocols, optimal dose selection, and sustaining long-term efficacy. Therefore, future research should prioritize developing safer, more efficient, and economically viable NK cell senescence intervention strategies to facilitate successful clinical translation.

## 5. Future Perspective

### 5.1 Defining the characteristics of NK cell senescence

To date, NK cell immunosenescence remains inadequately defined due to the absence of a uniform, specific set of markers. During NK cell development, cells at distinct developmental stages exhibit characteristic surface markers. Immature NK cells characteristically express CD122 alongside NCRs, such as NKp46, NKp30, and NKp44 [36, 47, 68, 71, 72, 74]. The migratory ability of NK cells is governed by chemokine receptors, including CXCR3, CX3CR1, and S1P5R [36]. These receptors play crucial roles in the localization and activity of NK cells. Longer-lived NK cells are characterized by elevated expression of surface CD57 molecules [7, 36]. As NK cells undergo senescence, the expression patterns of inhibitory receptors (e.g., KIRs, NKG2A) and activating receptors (e.g., NKG2D, NKp30) on their cell surface undergo modifications, reflecting alterations in their functional state [56, 72, 73]. In summary, monitoring surface molecular markers of NK cells enables the assessment of their maturity, functional status, and role in the immune system, while providing insights into the underlying senescence process.

Initially characterized features of senescent NK cells include diminished cytotoxic capacity and reduced ability to produce cytokines (e.g., IFN-γ, TNF-α); moreover, telomere length serves as a biomarker of cellular senescence. However, these characteristics do not constitute a definitive assay, and these biomarkers alone are insufficient to definitively characterize NK cell senescence. Notably, the cleaved granule gene FCGR3A (which encodes the Fcγ receptor IIIa, a high-affinity receptor for the Fc region of IgG, predominantly expressed on NK cells) and elevated proportions of KIR+ cells are indicative of terminally differentiated CD57+ NK cells, which exhibit cytotoxic properties while demonstrating limited proliferative responses to cytokines [30]. Another defining marker is S100A4, which progressively increases during NK cell maturation and reaches peak expression on CD57+ NK cells [30]. However, identifying a reliable panel of biomarkers to monitor NK cell senescence status and function remains a significant challenge in contemporary research, constrained by factors including detection feasibility, cost-effectiveness, accuracy, and sensitivity. Establishing a consensus on the comprehensive assessment of these markers would facilitate more accurate characterization of NK cell senescent states and provide a scientific foundation for developing interventions targeted at enhancing senescent NK cell function.

### 5.2 Subpopulation differentiation of senescent NK cells to be explored

Currently, limited research exists investigating the changes in subpopulation distribution during NK cell senescence. Several studies have begun to explore this issue, documenting an increase in the cytotoxic CD16+CD56dim NK cell subpopulation with higher CD57 expression and a simultaneous decrease in the immunomodulatory CD16-CD56bright NK cell subpopulation [26, 65]. The study of age-related changes in NK cell subsets has made substantial progress to date. However, it is crucial to distinguish between NK cell senescence per se and age-related changes in the immune system, as these represent related yet distinct concepts. NK cell senescence per se refers to the functional and phenotypic changes that occur in individual NK cells over time, as opposed to the broader age-related changes in the entire immune system. Changes in subpopulation distribution during NK cell senescence warrant investigation through observing NK cell behavior in long-term culture or under specific stress conditions.

### 5.3 Lack of effective measures to delay NK cell senescence

Investigating the intrinsic mechanisms of senescence in NK cells is crucial for understanding age-related changes in the immune system and developing targeted interventions to maintain NK cell function. Several knowledge gaps remain to be elucidated, including: the specific relationship between the TRAF/TAK1 signaling axis and TGF-β and NF-κB signaling [40]; the identification of receptors beyond CD158d that continuously trigger the senescent phenotype in human NK cells; and the optimal receptor targets for reversing NK cell senescence. Furthermore, the precise mechanisms by which HCMV induces NK cell hypofunction in immune senescence, as well as the cooperative role of MDSCs and Tregs in inducing NK cell senescence, require further elucidation. Addressing these questions will require diverse experimental techniques, including cell culture, flow cytometry, molecular biology analyses, and functional testing, to gain a detailed understanding of the intrinsic changes that occur in NK cells over time.

The role of cytokines in attenuating NK cell senescence warrants further investigation, as the direct effects of IL-21 and STAT3 activation on NK cells remain both ambiguous and controversial. Moreover, elucidating the involvement of Hsp90 and mTOR in regulating human telomerase activity in primary cells from patients with NK-cell lymphomas/leukemias, as well as the mechanisms by which the Akt/Hsp90/mTOR/S6K signaling pathway upregulates telomerase activity, is critical for future comprehensive studies [151]. Furthermore, whether the presence of SASP in NK cells is regulated by NFκB, mTOR, p38MAPK signaling, and IL-1 signaling through NLRP3 inflammasomes [40] still requires experimental verification.

### 5.4 Interactions between senescent NK cells and other cells are unclear

The interaction between senescent NK cells and other immune cells encompasses a complex network of interconnected signaling pathways and molecular mechanisms. Reduced NKp30 receptor expression in NK cells from healthy older adults has been demonstrated to play a critical role in regulating their interaction with DCs [3]. However, the precise mechanisms by which this reduction in NKp30 expression affects NK cell function remain to be fully elucidated. The effects of aging on NK cells and their interactions with T and B cells are fundamental to understanding the mechanisms underlying immune decline in the elderly. Current hypotheses suggest that senescence may alter the cytokine secretion profile of NK cells, subsequently influencing the proliferation, differentiation, and antibody production of T and B cells. Furthermore, the reduced cytotoxicity of senescent NK cells may induce a compensatory T cell-mediated immune response, thereby impacting both T and B cell functionality. Recent studies have demonstrated that NK cells may possess memory-like properties, which could significantly influence their interactions with adaptive immune cells, particularly memory T and B cells. Additionally, senescence may impair the immune surveillance capacity of NK cells, consequently leading to a weakened or delayed response of other immune cells to pathological challenges. Understanding the mechanisms governing these interactions will not only elucidate the alterations in the immune system during senescence but also provide essential insights for developing targeted immune interventions to improve the health of older adults. However, research in these areas remains in its infancy, and many of the specific mechanisms and their clinical implications warrant further investigation.

### 5.5 Controversies of NK cell senescence in anti-tumor immunity

The potential positive effect of NK cell senescence on antitumor immunity represents a highly complex and contentious topic in the field. Traditionally, NK cell senescence has been regarded primarily as a detrimental process due to its attenuation of both the anti-tumor and anti-viral capabilities of NK cells. However, the scientific community is increasingly acknowledging that senescence of the immune system, including NK cell senescence, may represent a more intricate phenomenon that encompasses aspects of immune regulation and microenvironmental adaptation. Further investigations may uncover previously unrecognized beneficial roles of these senescence processes and offer novel insights into the prevention and treatment of age-related diseases. The beneficial role of SASP in adaptation to normal vasculature and remodeling of healthy tissues during pregnancy provides evidence that the senescence process exists not only as a cancer prevention mechanism but also as a counterpoint to cancer development [14]. Age-associated NK cell immunosenescence contributes to both increased susceptibility to viral infections and elevated risk of cancer development. Furthermore, NK cell-mediated clearance of senescent cells diminishes during the senescence process, leading to an accumulation of senescent cells in tissues and organs, which subsequently impairs tissue homeostasis and function [13].

### 5.6 Relationship between NK cell senescence and antitumor drug sensitivity as an area for development

To date, there are no direct studies on the relationship between NK cell senescence and antitumor drug sensitivity; however, analogies to T cell senescence may provide valuable insights for investigating NK cell senescence. Studies of the human immune system have demonstrated that the immune state does not manifest as a segregated hierarchical structure but rather exhibits a continuous spectrum, with immune senescence-associated features serving as the dominant factor driving changes. Consequently, the "biological age" of the immune system may differ substantially even among individuals of the same chronological age [150]. The use of combinatorial biomarkers to identify senescent cells for antitumor immunosenescence therapy has been extensively investigated [29, 151]. In cardiovascular disease [150], leukemia, and melanoma [151], antitumor drug sensitivity is enhanced by modulating T cell metabolic reprogramming, antagonizing senescent T cells, or promoting T cell proliferation and infiltration [152]. Our current understanding of the effects of NK cell senescence on antitumor drug sensitivity remains limited. The specific mechanisms by which NK cell senescence affects antitumor drug sensitivity have not been adequately explored. Future research should primarily focus on the antitumor therapeutic sensitivities of immune checkpoint inhibitors [90] and CAR-NK [153], as T cell senescence plays a central role in antitumor drug sensitivities. The efficacy of CAR-T therapies depends on their fate determination and interaction with cancer cells in the tumor microenvironment (TME), which is also closely linked to the metabolic adaptation of NK cells [90].

Developing effective immune checkpoint inhibitors and CAR-NK therapies requires robust strategies to overcome antitumor therapy-associated NK cell senescence. At the macro level, specific clinical conditions (e.g., concomitant disease, concomitant medication use, and decline in physiologic function), coupled with the phenomenon of immune senescence in older patients, may impact both the efficacy and potential toxicity of immune checkpoint inhibition therapy [90]. At the microscopic level, researchers are actively employing large-scale RNA sequencing techniques to identify specific biomarkers that reliably indicate senescent cell prevalence. Meanwhile, single-cell RNA (scRNA) sequencing, a sophisticated technique capable of analyzing the complete transcriptome of a single cell, has been utilized to reveal the unique transcriptional signatures of rare senescent cells in vivo [158, 159]. At this stage of research, investigations into the anti-tumor immune sensitivity of senescent NK cells should incorporate other major cells in the TME, despite differences in senescence mechanisms and variable effects of anti-aging drugs across different cell types. In a mouse model of aging, genetically engineered IL-37 expression effectively promoted functional recovery of senescent B-cell progenitors and abrogated their selective advantage over B-cells harboring oncogenic mutations, thus preventing leukemogenesis [151]. Notably, IL-37 application led to a significant decrease in cell surface PD-1 expression and a marked reduction in the activation levels of PD-1 and TNF-α signaling pathway downstream genes (TMEM16F, GM130, PD-1, and SHP2) [151]. These findings offer promising avenues for future research investigations. Understanding NK cell senescence and its impact on the cells' capacity for tumor surveillance and clearance holds significant potential for improving treatment outcomes in elderly cancer patients.

Despite extensive research on the relationship between T-cell senescence and antitumor drug sensitivity, numerous aspects remain poorly defined, highlighting substantial gaps in the study of NK-cell senescence. The efficacy and widespread adoption of CAR-T cell therapy [160] face multiple challenges, including limited in vivo persistence, restricted migration of CAR-T cells to tumor sites, extensive tumor heterogeneity and their adaptive evolution leading to non-specific targeting, and T-cell dysfunction or depletion resulting from prolonged antigen exposure or immunosuppressive effects within the TME [90]. Moreover, corticosteroids serve as key therapeutic agents in immunotherapy, especially during or after checkpoint inhibition therapy, for managing possible immune-related adverse events (irAEs) [140]. Future studies should focus on precisely evaluating how autoantibodies and immune system senescence influence the incidence of irAEs as well as the responsiveness and toxicity of anti-PD-L1 therapy in elderly patients [73, 86, 161, 162]. In summary, while NK cell senescence induced by antitumor drug therapy is well documented, this field still necessitates substantial research input. The relationship between NK cell senescence and tumor drug sensitivity encompasses multiple dimensions, including cytokine regulation, immune checkpoint expression, restoration of cellular function, and patient-specific clinical characteristics. As knowledge in this field advances, the development of therapeutic strategies targeting the senescent immune system to enhance tumor treatment outcomes has emerged as a critical research priority.

**Figure 5.**
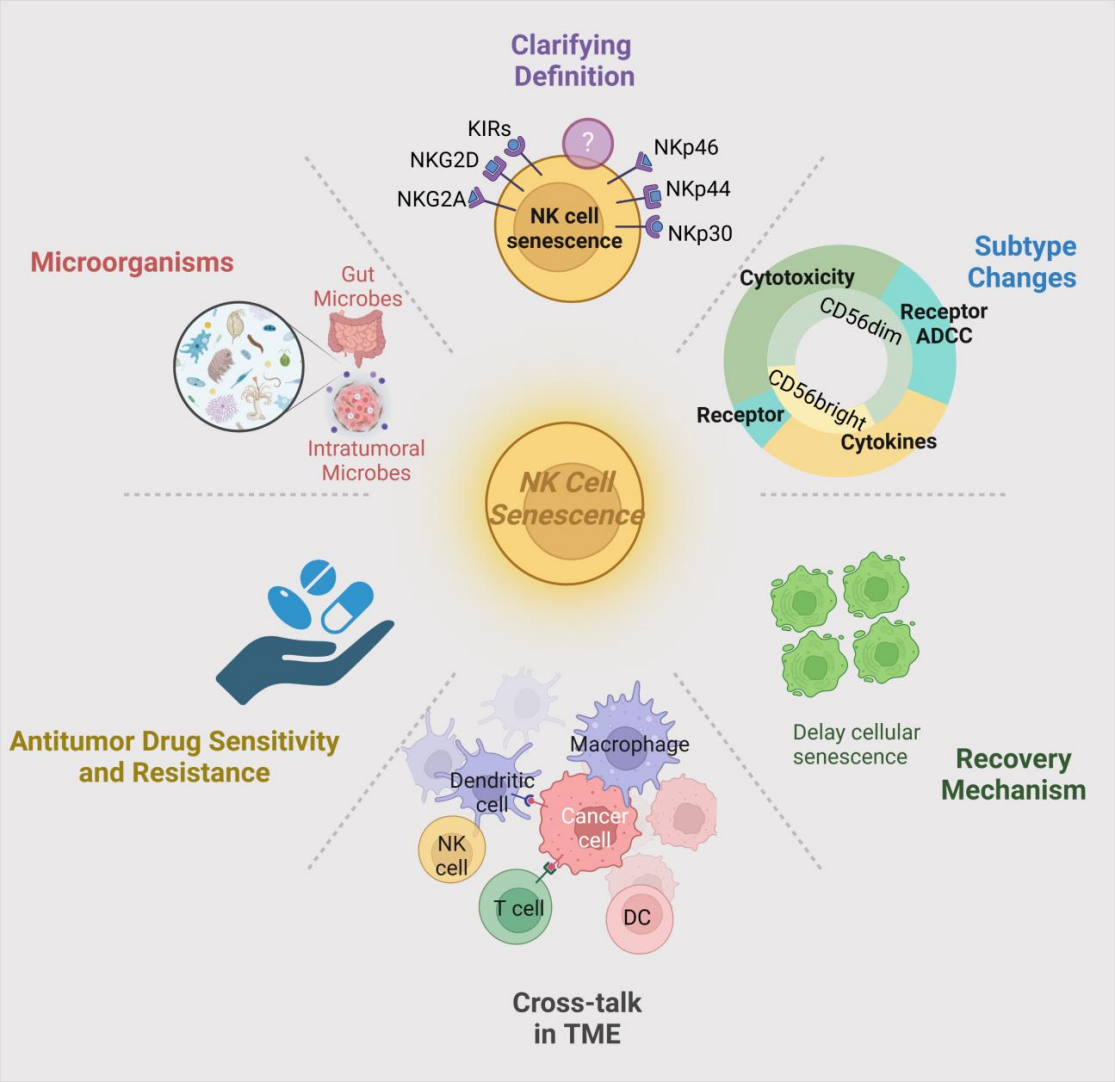
**Open questions in NK cell senescence research**. The diagram concisely outlines key areas of ongoing research in NK cell senescence, including senescence characteristics, subpopulation differentiation, recovery mechanisms, cell-cell interactions, the relationship between NK cell senescence and antitumor drug sensitivity, and interactions with intratumoral microorganisms. Top: Immature NK cells characteristically express CD122 and natural cytotoxicity receptors (NCRs), including NKp46, NKp30, and NKp44. As NK cells undergo senescence, the expression patterns of inhibitory receptors (e.g., KIRs, NKG2A) and activating receptors (e.g., NKG2D, NKp30) on their surface are altered. Identifying a comprehensive panel of reliable biomarkers to monitor NK cell senescence and function remains a critical challenge in contemporary research. Above right: The figure comprises two concentric circles. The inner circle depicts the classification of the two main NK cell subpopulations, while the outer circle illustrates the characteristics and functions of NK cells. The larger green portion of the inner circle, relative to the yellow portion, indicates that the CD56dim subpopulation is more abundant than the CD56bright subpopulation. The overlap between the outer and inner circles indicates that both subpopulations possess these functions, albeit to varying degrees. For instance, although both CD56dim and CD56bright subpopulations exhibit cytotoxicity, the CD56dim subpopulation demonstrates superior cytotoxic potential, as illustrated in the figure. Similarly, while both CD56dim and CD56bright subpopulations secrete cytokines, the CD56bright subpopulation exhibits enhanced capacity for cytokine production compared to the CD56dim subpopulation. The bottom right panel emphasizes the importance of investigating mechanisms underlying NK cell senescence to elucidate immune system evolution and to maintain NK cell function through targeted interventions. Future investigations should prioritize identifying effective strategies to attenuate NK cell senescence. The bottom panel underscores that elucidating cellular interaction mechanisms not only enhances understanding of age-related immune system changes but also highlights the necessity for further investigation and clarification of specific mechanisms and their effects. Initial studies should examine the interactions among NK cells, T cells, macrophages, and B cells. The lower left panel demonstrates that the precise mechanisms through which NK cell senescence influences antitumor drug sensitivity remain largely unexplored. Future research should focus on investigating the impact of NK cell senescence on the efficacy of immune checkpoint inhibitors and chimeric antigen receptor (CAR)-NK cell-based antitumor therapies. The top left panel indicates that the interplay between microbiota (both intratumoral and gut) and NK cell senescence constitutes a promising avenue for future research. Studies should explore the potential for modulating microbiota composition as an approach to delay or reverse NK cell senescence. This figure was created using tools provided by Biorender.com (accessed on May 30, 2024).

### 5.7 Relationship between microorganisms (within the tumor) and NK cell senescence in TME

The relationship between the microbiota within the TME or gut and NK cell senescence remains an underexplored area of research with the potential to uncover novel links between immune system senescence, tumor progression, and therapeutic response. Imbalances in the gut microbiota have been implicated in age-related diseases, and these imbalances may impair host immune system function, including NK cell activity [66, 163]. Notably, aging-associated immune system changes, such as T-cell senescence, dysfunctional B-cells, NK cells, and neutrophils, play a key role in maintaining the host's symbiotic relationship with these microbes [164, 165]. With advancing age, this decline in immunosurveillance may result in profound and widespread effects on host health and immune function. Furthermore, reducing levels of TNFα, a key cytokine associated with low-grade inflammation, either through genetic manipulation or through the use of targeted antibodies, can effectively mitigate the imbalance of gut microbes associated with senescence and the ensuing state of systemic low-grade inflammation [163, 166-168]. This strategy highlights the potential to maintain gut microbial homeostasis and regulate systemic inflammation levels by modulating specific inflammatory mediators (especially early in life) [169, 170], thereby offering a possible pathway to delay or reverse age-related physiological changes [133].NK cells represent a key component of the innate immune system and play a crucial role in combating tumors and infectious diseases [44, 171]. However, as the senescence process progresses, NK cell function significantly declines, impairing their cytotoxic activity and capacity to produce cytokines [62]. If alterations in the microbiota, particularly within the TME or the gut, can directly or indirectly influence NK cell senescence, this finding would establish a basis for developing novel therapeutic strategies that could enhance NK cell function by modulating the microbiota to combat cancer and infections while attenuating immunosenescence. Currently, research investigating the specific effects of microbiota on NK cell senescence remains scarce, and additional experimental and clinical studies are essential to explore this field. In conclusion, the relationship between the microbiota and NK cell senescence represents a promising research frontier, and future studies should focus on elucidating how specific compositional alterations in the microbiota correlate with the decline in NK cell function and whether NK cell senescence can be attenuated or reversed by targeted modulation of the microbiota composition. These findings may provide valuable insights into the mechanisms underlying immunosenescence and facilitate the development of novel anticancer and immunomodulatory strategies, ultimately leading to innovative approaches for managing elderly health and treating age-related diseases. The future perspective for NK cell senescence is summarized in Figure 5.

### Summary

NK cells, a subset of lymphocytes that are primarily involved in innate immunity, play a crucial role in generating effective and durable antitumor responses. NK cell immunosenescence may impair the crosstalk between the innate and adaptive immune systems; however, limited studies have investigated the effects and underlying mechanisms of NK cell senescence within the TME. In this review, we highlight recent advances in defining and characterizing NK cell senescence, elucidate the mechanisms by which the TME induces NK cell senescence, and discuss the implications for immunotherapy. Nevertheless, these studies have significant limitations and raise critical questions that warrant further investigation. First, the specificity and consistency of senescence markers remain debatable in the context of NK cells. Moreover, the direct causal relationship between senescence and functional decline requires further clarification, and the characterization of distinct NK cell subpopulations warrants additional exploration. Furthermore, the TME accelerates the senescence process of NK cells by inducing the production of immunosuppressive cells and cytokines, thereby compromising their efficacy in tumor control. Future studies should systematically examine the complexity of the TME and its effects on NK cell function. For instance, the collective influence of multiple factors within the TME, such as persistent inflammation, inhibitory cytokines, and suppressive immune cells, on NK cell function remains incompletely understood. This necessitates an in-depth exploration of how these factors synergistically promote NK cell senescence and consequently impair their role in tumor immunosurveillance.
